# Quinolinate as a Marker for Kynurenine Metabolite Formation and the Unresolved Question of NAD^+^ Synthesis During Inflammation and Infection

**DOI:** 10.3389/fimmu.2020.00031

**Published:** 2020-02-21

**Authors:** John R. Moffett, Peethambaran Arun, Narayanan Puthillathu, Ranjini Vengilote, John A. Ives, Abdulla A-B Badawy, Aryan M. Namboodiri

**Affiliations:** ^1^Departments of Anatomy, Physiology and Genetics and Neuroscience Program, Uniformed Services University Medical School, Bethesda, MD, United States; ^2^The Center for Brain, Mind, and Healing, Samueli Institute, Alexandria, VA, United States; ^3^Independent Consultant, Cardiff, United Kingdom

**Keywords:** quinolinic acid, indoleamine 2,3-dioxygenase, kynurenine pathway, PARP, cell motility, foam cells, HSP90, ERM proteins

## Abstract

Quinolinate (Quin) is a classic example of a biochemical double-edged sword, acting as both essential metabolite and potent neurotoxin. Quin is an important metabolite in the kynurenine pathway of tryptophan catabolism leading to the *de novo* synthesis of nicotinamide adenine dinucleotide (NAD^+^). As a precursor for NAD^+^, Quin can direct a portion of tryptophan catabolism toward replenishing cellular NAD^+^ levels in response to inflammation and infection. Intracellular Quin levels increase dramatically in response to immune stimulation [e.g., lipopolysaccharide (LPS) or pokeweed mitogen (PWM)] in macrophages, microglia, dendritic cells, and other cells of the immune system. NAD^+^ serves numerous functions including energy production, the poly ADP ribose polymerization (PARP) reaction involved in DNA repair, and the activity of various enzymes such as the NAD^+^-dependent deacetylases known as sirtuins. We used highly specific antibodies to protein-coupled Quin to delineate cells that accumulate Quin as a key aspect of the response to immune stimulation and infection. Here, we describe Quin staining in the brain, spleen, and liver after LPS administration to the brain or systemic PWM administration. Quin expression was strong in immune cells in the periphery after both treatments, whereas very limited Quin expression was observed in the brain even after direct LPS injection. Immunoreactive cells exhibited diverse morphology ranging from foam cells to cells with membrane extensions related to cell motility. We also examined protein expression changes in the spleen after kynurenine administration. Acute (8 h) and prolonged (48 h) kynurenine administration led to significant changes in protein expression in the spleen, including multiple changes involved with cytoskeletal rearrangements associated with cell motility. Kynurenine administration resulted in several expression level changes in proteins associated with heat shock protein 90 (HSP90), a chaperone for the aryl-hydrocarbon receptor (AHR), which is the primary kynurenine metabolite receptor. We propose that cells with high levels of Quin are those that are currently releasing kynurenine pathway metabolites as well as accumulating Quin for sustained NAD^+^ synthesis from tryptophan. Further, we propose that the kynurenine pathway may be linked to the regulation of cell motility in immune and cancer cells.

## Introduction

Considering the breadth of studies linking the tryptophan catabolite quinolinate (Quin) to neurotoxicity and neurological disorders, it is perhaps ironic that the earliest studies on Quin tied it to a critical biological function as a precursor for nicotinamide adenine dinucleotide (NAD^+^) synthesis (1, 2). The irony lies in the rarity of subsequent studies investigating the role of Quin in the biosynthetic pathway from tryptophan to NAD^+^ in different cell types, under various physiological and pathological circumstances. Many studies into Quin over the last several decades have focused on its connections to neurological disorders [reviewed in (3–9)]. More recently, attention has also turned to the immunomodulatory and immunosuppressive effects of kynurenine metabolites (10, 11). To this day, the extent and criticality of the contribution of tryptophan catabolism to NAD^+^ synthesis in cells of the immune system during health and disease remain mostly unexplored (12). Yet, it is becoming increasingly clear that NAD^+^ requirements in certain cell types can be elevated during inflammation, injury, and infection, and that this is critical for proper immune cell responsiveness (13–16). As such, the contribution of the kynurenine pathway to sustaining NAD^+^ levels in the immune system during various challenges may turn out to be substantial in certain cell types.

**Figure 1:**
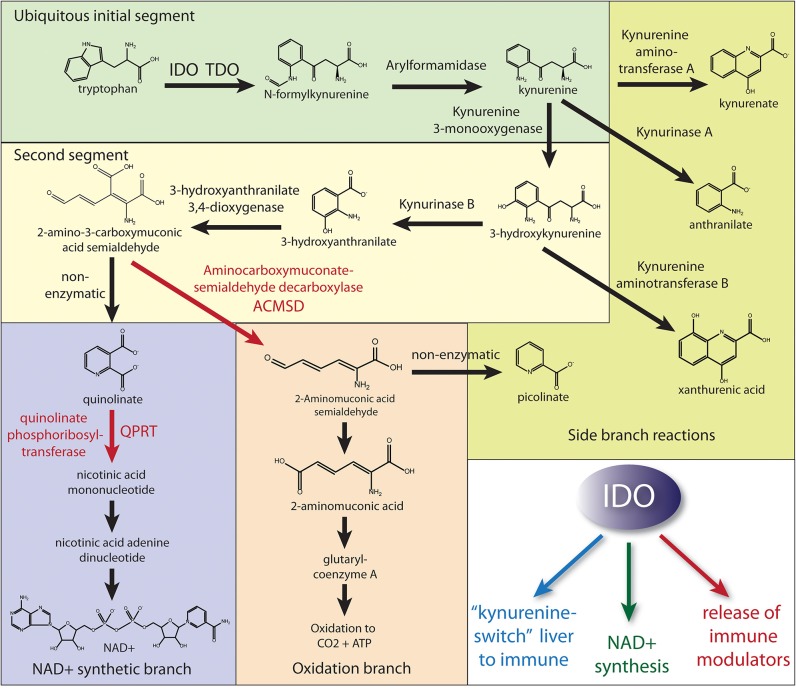
Simplified diagram of the kynurenine pathway of tryptophan catabolism. Most cell types can initiate the kynurenine pathway via either TDO or IDO to produce kynurenine (initial segment of tryptophan metabolism). Hepatocytes have the full complement of enzymes to either produce NAD^+^ or fully oxidize tryptophan to CO_2_. Numerous cell types, including many of the immune system, express the enzymes through the NAD^+^ synthetic branch. However, in order for Quin to build up in some immune cells during an immune response, the activities of the enzymes aminocarboxymuconate semialdehyde decarboxylase (ACMSD) and quinolinate phosphoribosyltransferase (QPRT) must be restricted to slow further metabolism to either NAD^+^ or oxidation to CO_2_. The fate of stockpiled Quin in those immune cells remains uncertain, but it is likely that both NAD^+^ synthesis and oxidation to yield energy are employed by various cells of the immune system during an immune response. Also, these cells may be releasing upstream metabolites. As such, upregulation of QPRT activity (red arrow) would be the rate-limiting factor for further metabolism to NAD^+^ when needed, and we propose this branch is predominantly utilized in cells of the immune system following IDO activation. In contrast, the activity of ACMSD would control the oxidative branch throughput for energy derivation. The three primary functions of IDO activation are (1) the extra-hepatic production of kynurenine, which is released for uptake by cells of the immune system thus diverting tryptophan metabolism to the immune system, (2) the production of NAD^+^ in cells of the immune system for the PARP reaction to DNA damage and other critical functions in immune cells, and (3) the production and release of immune modulating metabolites to regulate the immune response, especially T cell responsiveness. NMNAT, nicotinamide mononucleotide adenylyltransferase; NADSYN1, NAD synthetase 1.

One possible reason for the paucity of studies into NAD^+^ synthesis from tryptophan in health and disease is that modern diets provide the majority of the requisite precursors for NAD^+^ synthesis in the form of vitamin B3 (niacin; including both nicotinic acid and nicotinamide). However, when the diet does not provide sufficient niacin or the immune system is challenged, tryptophan catabolism is required to maintain sufficient NAD^+^ synthesis. The pathway of tryptophan metabolism to NAD^+^ is known as the *kynurenine pathway* because one of the early metabolites in this catabolic pathway is kynurenine (Figure 1). Two physiologically distinct, rate-limiting enzymes initiate tryptophan catabolism to NAD^+^; tryptophan 2,3-dioxygenase (TDO) and indoleamine 2,3-dioxoygenase (IDO) [reviewed in (17)]. TDO is expressed extensively in hepatocytes, as well as in many other cell types throughout the body. IDO is expressed extensively in cells of the immune system, but is also found in many other cell types. The enzyme quinolinate phosphoribosyltransferase (QPRT) catalyzes the formation of nicotinic acid mononucleotide from Quin and 5-phosphoribosyl-1-pyrophosphate, fueling NAD^+^ synthesis. Because NAD^+^ is a cofactor in numerous redox and other important cellular reactions, some of which become substantially increased during inflammation and infection, the synthesis of NAD^+^ may be enhanced when the immune system responds to challenges. Despite these facts, the importance of Quin in the synthesis of NAD^+^ during the immune system's responses to infections, cancer, or injury remains much more poorly understood than its neurotoxic effects.

The dramatic increase in tryptophan catabolism via IDO during immune system responses is evolutionarily conserved (18–21), indicating its pro-survival value. Yet, the precise nature of the benefits that the kynurenine pathway confers on fitness is a matter of ongoing debate (7, 9, 16, 22, 23). Known functions of the kynurenine pathway include the production of immune-regulatory metabolites, especially immunosuppressive ones, as well as NAD^+^ synthesis. Both of these functions intersect during an immune response where immunosuppression and increased NAD^+^ availability are necessary for a successful resolution of infection or injury. The surge in IDO expression during infection or after experimental immune stimulation is accompanied by an increase in kynurenine metabolites, including Quin, in blood and in tissues. Further, Quin levels increase in specific cell populations as shown by Quin immunohistochemistry. Specialized fixation and antibody purification methods permit the visualization of Quin in cells with very high sensitivity and specificity (24–27). These methods allow detailed visualization of cells that synthesize Quin and have demonstrated that specific cells of the immune system accumulate high intracellular concentrations of Quin. In the current study, we investigated this phenomenon by stimulating the immune system with either lipopolysaccharide (LPS) or pokeweed mitogen (PWM). We delivered LPS to the hippocampus of rats and administered PWM intraperitoneally (IP) to gerbils. We then used specialized immunohistochemistry to follow the course of Quin buildup in cells in the brain, spleen, and liver.

## Materials and Methods

The digital images presented here were generated from archival slides prepared previously and include material from both published (25–29) and unpublished studies. The methods used for the production, purification, and characterization of the polyclonal Quin antibodies have been described previously (26). The carbodiimide-based immunohistochemical methods have also been described (30). The Quin immunohistochemistry methods developed by us have been validated independently by another laboratory, using the same protocols (31). Imaging was done using an Olympus BX-51 microscope and Olympus DP-71 digital camera. Images were acquired using Image Pro Plus software (ver. 7.1; Media Cybernetics). Chemicals were from Sigma (St. Louis, MO). Horseradish peroxidase (HRP)-coupled goat anti-rabbit secondary antibodies were purchased from Kirkegaard and Perry (Gaithersburg, MD). Avidin–biotin complex kits (Vectastain Elite), normal goat serum (NGS), biotinylated GSL I-B_4_, and HRP-labeled streptavidin were from Vector (Burlingame, CA). Polyclonal antibodies to GFAP (AB1980) were from Chemicon (Temecula, CA).

### Immune Stimulation Models

Here, we compare the Quin-immunoreactivity (Quin-IR) response in two models of immune stimulation including (1) IP PWM injection in gerbils where we examined the Quin-IR response at 24 h and (2) LPS injection into the hippocampus of rats, examined at multiple time points. LPS and PWM act on immune cells via different receptor systems. For example, in Kupffer cells (hepatic macrophages), LPS activates TRL4 receptors, whereas PWM activates the complement system via cleavage of C3 (32). Both agents induce strong IDO activity in the immune system. Using these two models, we compared the Quin-IR response during peripheral immune stimulation vs. central nervous system immune stimulation.

We also examined the effects of L-kynurenine administration on protein expression in the spleen of mice. This model engaged the kynurenine pathway by bypassing the rate-limiting enzymes, IDO and TDO, providing a view into the effects of increased kynurenine pathway throughput on protein expression in the spleen in the absence of immune stimulation. All experimental protocols were approved by the Uniformed Services University of the Health Sciences (USUHS) animal care and use committee.

### Intracerebral Injections of LPS

For these animals, under halothane anesthesia, a 30-gauge stainless steel cannula was inserted into the dorsal hippocampus (from bregma: AP = −4.2, Midline = +1.8, DV = −2.9), and 2 μl of sterile saline containing 4 μg of LPS from *Salmonella arbortus equi* was injected over a 10-min period. The cannula was withdrawn 30 min later, the incision closed, and the animals allowed to recover in a heated cage. Animals were perfused, as described below, from 1 to 30 days after LPS injection, with 2 to 3 animals per time point.

### Intraperitoneal Injection of PWM

Methods for gerbils given IP injections of PWM have been previously described (28). Animals were anesthetized and perfused via carbodiimide fixation (described below) 24 h after PWM administration.

### Animal Perfusions

The animals were anesthetized with pentobarbital and perfused transcardially with 400 ml of an aqueous solution of 6% 1-ethyl-3 (3-dimethylaminopropyl) carbodiimide hydrochloride, 6% DMSO, and 1 mM N-hydroxysuccinimide warmed to 37°C (26, 30). This fixative is required for Quin immunohistochemistry. The brains, spleens, and livers were removed to a solution of 4% paraformaldehyde in 100 mM phosphate buffer at pH 8 for 24 h. The tissues were then passed through a series of 10, 20, and 30% sucrose solutions in PBS.

### Immunohistochemistry

The immunohistochemical methods have been described in detail previously (26, 27, 30). Briefly, tissues were cryo-sectioned at a thickness of 20 microns and were collected serially in groups of 5, and placed in 24-well tissue culture plates in the same solution. Endogenous peroxidase was blocked with a 50:50 mixture of methanol and water containing 1% H_2_O_2_ for 30 min with agitation. Tissue sections were incubated overnight with the Quin antibodies, diluted 1:10,000, using constant rotary agitation. The antibodies were visualized by the avidin–biotin complex method with HRP as the enzyme marker (Vectastain Elite, Vector Labs). The sections were incubated with the biotinylated secondary antibody and avidin–biotin–peroxidase complex solutions for 90 min each, and then developed with diaminobenzidine and urea peroxide as chromogen and substrate (Sigmafast DAB tablets, Sigma) for 10–15 min. Lectin histochemistry with *Griffonia simplicifolia*-B_4_ lectin (GSL-IB_4_) and double staining with Quin was done as described earlier (27).

## 2D-DIGE Proteomics and LC-MS/MS

We used two-dimensional, differential image gel electrophoresis (2D-DIGE) to investigate the effects of kynurenine on protein expression in the spleen in the absence of inflammation, infection, or injury, and therefore in the absence of increased IDO activity. We administered high-dose L-kynurenine to normal mice and used fluorescent 2D proteomics to look for changes in protein expression in the spleen. Male C57 mice weighing between 20 and 25 g were used for these studies (Charles River Labs, Wilmington, MA). Animals were housed in the USUHS animal facility on a 12/12 h light–dark cycle, with *ad libitum* access to food and water. We compared a single high-dose IP injection of kynurenine to administration of kynurenine in drinking water for 48 h. In group 1, three experimental animals were given a single IP injection of 300 mg/kg of L-kynurenine diluted in sterile saline, whereas three control animals were given a single IP injection of sterile saline 8 h before sacrifice. In group 2, three mice were given free access to drinking water containing 5 mM kynurenine for 48 h before sacrifice, and three control animals were given access to plain tap water.

### 2D Gels

Eight hours after kynurenine injections or after 48 h of prolonged administration in drinking water, mice were anesthetized with pentobarbital (300 mg/kg) and killed by decapitation. Spleens were removed and frozen immediately on dry ice. The 2D-DIGE proteomics and mass spectroscopy were done at the Windber Research Institute (Windber, PA) as previously described (33). Spleen tissues were homogenized in an ice-cold lysis solution (2 M thiourea, 6 M urea, 4% CHAPS, 1% NP-40, 5 mM magnesium acetate, and 30 mM Tris–HCl at pH 8.5). Gels were run using the 2D-DIGE method (Amersham Biosciences). Experimental and control animals were paired randomly, and the experimental and control protein samples were labeled separately with a red or green fluorescent dye. A control mixture of the two samples was made with equal amounts of protein from each of the paired animals, and this was labeled with a yellow fluorescent dye. All three labeled protein samples from each pair of animals were mixed, and each mixture was subjected to 2-D gel electrophoresis (34). Gels were loaded with 50-μg samples, and the first dimension was run with an immobilized pH gradient from pH 3 to 10 (Immobiline dry strips). The second dimension was run on a 12% SDS-PAGE gel.

### MALDI and LC/MS/MS

Gel image analysis was done using the DeCyder software package. Statistical analysis was done using the DeCyder biological variation analysis module (Amersham) and Student's *t*-test was used to determine differential expression (35). The destaining, digestion, extraction, and MALDI (matrix-assisted laser desorption/ionization) sample preparation were carried out robotically in an enclosed unit according to the manufacturer's instructions (Amersham). MALDI peptide mass fingerprinting was carried out on an Ettan Pro MALDI-ToF mass spectrometer (Amersham Biosciences) operating in reflectron mode. Sensitivity of the system allowed identification of 40 fmol of protein. The mass spectra were internally calibrated using trypsin autodigestion peaks at m/z 842.542 and 2211.109 to give a mass accuracy of better than 100 ppm. Protein identification by peptide mass fingerprinting was performed using the MASCOT search engine and the NCBInr protein database.

## Results

### Intraperitoneal Injection of PWM in Gerbils

Our original short communication on PWM administration in the gerbil showed several images of the Quin-IR changes in the spleen and brain (28). Here, we provide a more detailed view of the observed changes. PWM is a potent immune stimulant that induces a large increase in Quin production (36). Further, PWM is one of the more potent inducers of INF-γ release (37). IP injection of PWM in gerbils elicited a very strong peripheral kynurenine pathway response in the spleen and liver, but no detectable increase in Quin-IR in brain parenchyma.

### Quin-IR in Gerbil Brain

**Figure 2:**
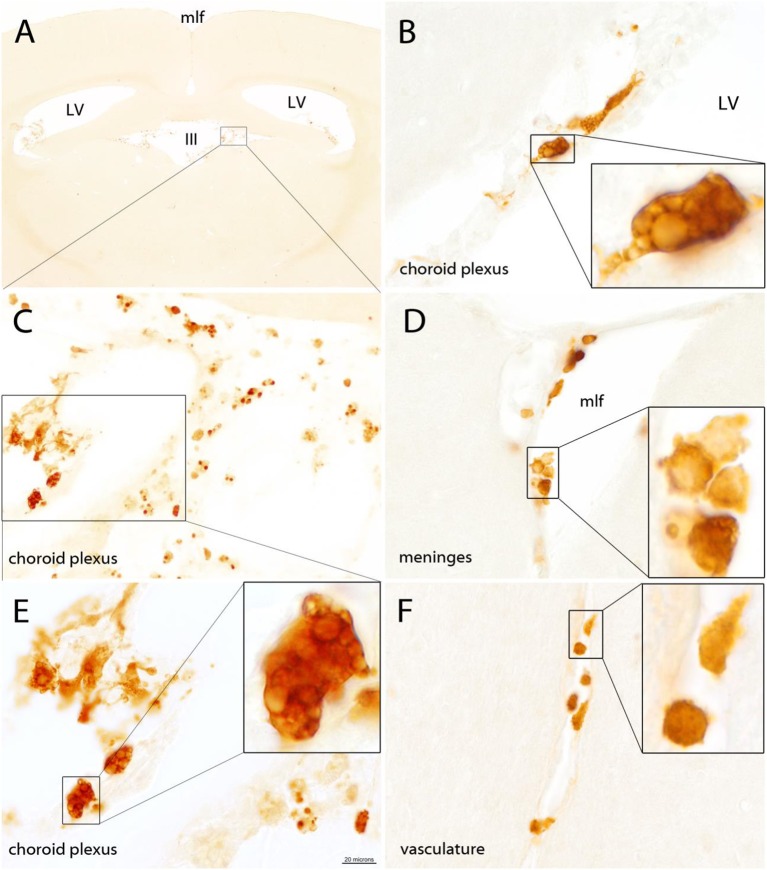
The gerbil brain is shown stained for Quin 24 h after IP injection of PWM (500 μg). No Quin-IR cells were observed within the brain parenchyma **(A)**. The majority of Quin-IR cells were found in the choroid plexus **(B,C,E)**. Additional Quin-IR cells were observed in the meninges, for example, at the apex of the medial longitudinal fissure **(D)**. Quin-IR cells were also observed adherent to the interior surfaces of the brain vasculature **(F)**. Quin-IR cells ranged widely in morphology, and some appeared relatively unstained but contained Quin-IR phagosomes, which may have been phagocytized debris from apoptotic cells. Others appeared similar to so-called “foam cells,” which are activated macrophages filled with lipid-containing vesicles (insets in **B,E**). Bar in **(E)**, 320 μm in **(A)**, 40 μm in **(C)**, 20 μm in **(B,D,E,F)**, and 6 μm in the insets. III, third ventricle; LV, lateral ventricles; mlf, medial longitudinal fissure.

Quin-IR was absent from brain parenchyma in the gerbil 24 h after peripheral immune stimulation with PWM (Figure 2A). Careful examination of many brain tissue sections from three gerbils given PWM demonstrated that Quin-IR cells from the periphery were excluded from brain parenchyma. However, Quin-IR cells were observed within the vasculature of the brain (Figure 2F). Quin-IR cells were adherent to the vessel surface in arteries and veins of all diameters, including capillaries. Quin-IR cells were also present in the meninges (Figure 2D). A modest but consistent Quin-IR reaction was observed in the choroid plexus where Quin-IR cells were common in the PWM-treated group (Figures 2B,C,E). Some of the Quin-IR cells in the choroid plexus appeared to be foam cells, which are macrophages that are filled with lipid-laden vesicles [reviewed in (38)].

### Quin-IR in Gerbil Spleen

**Figure 3:**
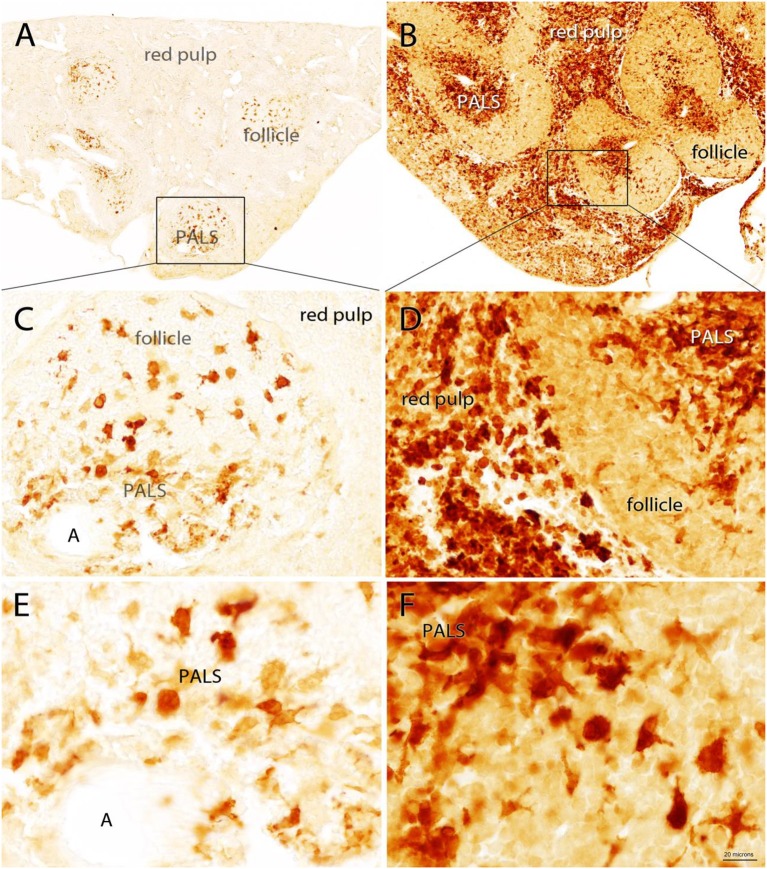
Quin-IR in the gerbil spleen 24 h after IP injection of saline **(A,C,E)** or PWM **(B,D,F)**. Quin-IR was low in the saline group, with most stained cells being present in the periarteriolar lymphoid sheaths (PALS) where antigen presentation occurs between dendritic cells and T cells. Cells in the red pulp were unstained or very lightly stained. In contrast, after PWM administration, Quin-IR was dramatically increased in both the red pulp and PALS. The core of the PALS was strongly stained, suggesting that the Quin-IR cells were dendritic cells. Intense Quin-IR was observed in the red pulp of PWM stimulated gerbils. A in panels **(C,E)**—arteriole. Bar in **(F)**, 200 μm in **(A,B)**, 40 μm in **(C,D)**, and 20 μm in **(E,F)**.

Moderate splenomegaly was observed in gerbils 24 h after IP administration of 500 μg of PWM. Measurements of the longest dimensions in width and height showed that cross-sectional dimensions of spleen slices were increased 25% in width and 48% in height 24 h after PWM administration (see Supplementary Figure 1). A robust increase in Quin-IR was present in the red pulp and in the central region of the periarteriolar lymphoid sheaths (PALS) in gerbils given PWM (Figure 3). In the saline-injected control group, Quin-IR in the spleen was generally very low, and was present in large, irregularly shaped cells in the white pulp (PALS and follicles in Figures 3A,C,E). Twenty-four hours after PWM administration, Quin-IR increased dramatically in the red pulp and in the PALS region of the white pulp (Figures 3B,D,F). Quin-IR in the cells in the B-cell-rich follicles also increased, but to a lesser degree than in the PALS and red pulp. These results show that in gerbils, as with other species, many cells of the immune system are capable of accumulating significant concentrations of Quin in their cytoplasm in response to potent immune stimulants.

### Quin-IR in Gerbil Liver

**Figure 4:**
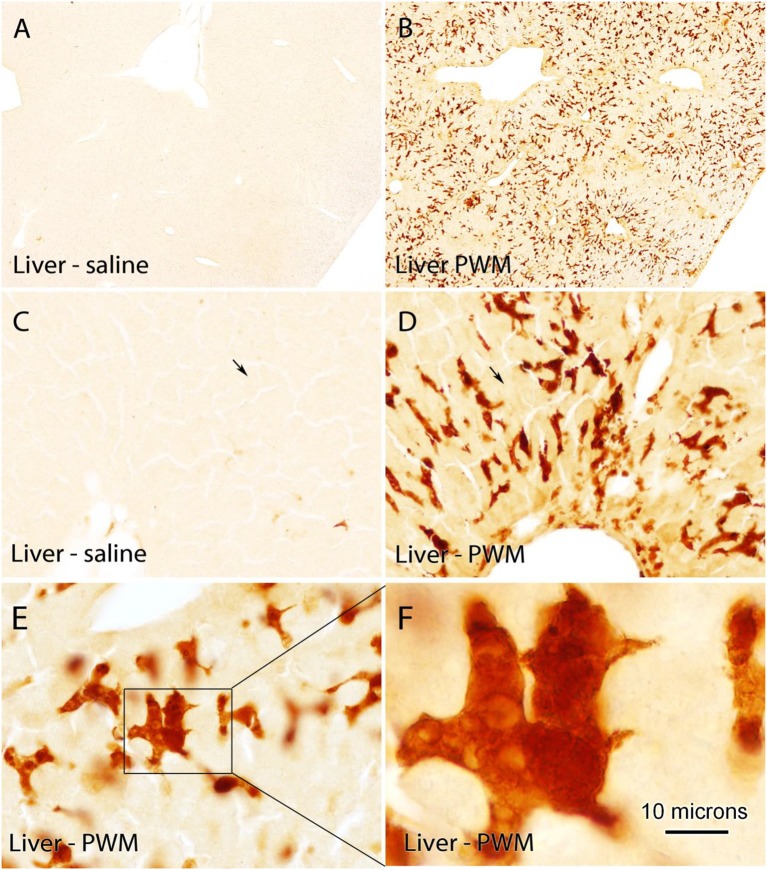
Quin-IR in the gerbil liver 24 h after IP PWM injection (500 μg). Quin-IR was very low in the liver of saline-injected gerbils **(A,C)**. However, 24 h after PWM injection, very strong Quin-IR was observed in cells throughout the liver **(B)**. Morphologically, the cells were identified as resident hepatic macrophages known as Kupffer cells **(D,E,F)**. Quin-IR increased slightly in hepatocytes in the PWM-stimulated liver (arrows in **C,D**), but staining was substantially lower than in the presumptive Kupffer cells. Bar in **(F)**, 370 μm in **(A,B)**, 74 μm in **(C,D)**, 37 μm in **(E)**, and 10 μm in **(F)**.

Quin-IR in the liver of gerbils in the control group given saline was very low (Figures 4A,C). The vast majority of cells were unstained. Occasional cells at the margin of sinuses were lightly stained. Twenty-four hours after PWM challenge, Quin-IR in the liver was dramatically increased in cells with the morphology of activated Kupffer cells (Figures 4B,D–F), whereas most hepatocytes appeared very lightly stained as compared with controls (arrows in Figures 4C,D). Additionally, based on location and morphology, some of the Quin-IR cells appeared to be hepatic dendritic cells. At high magnification, many of the strongly stained Kupffer cells contained numerous visible inclusions that could be phagosomes or phagolysosomes (Figures 4D–F). Kupffer cells are resident hepatic macrophages (39), and it has been shown that they respond to IFN-γ by activation of IDO and production of kynurenine (40). These investigators reported that activated Kupffer cells inhibited T cell proliferation and induced apoptosis of allogeneic T cells. Our results show that Kupffer cells are capable of catabolizing tryptophan to the level of Quin, which can then be used for NAD^+^ synthesis during an immune response.

## LPS Injection Into Rat Hippocampus

### Quin-IR in Rat Spleen

**Figure 5:**
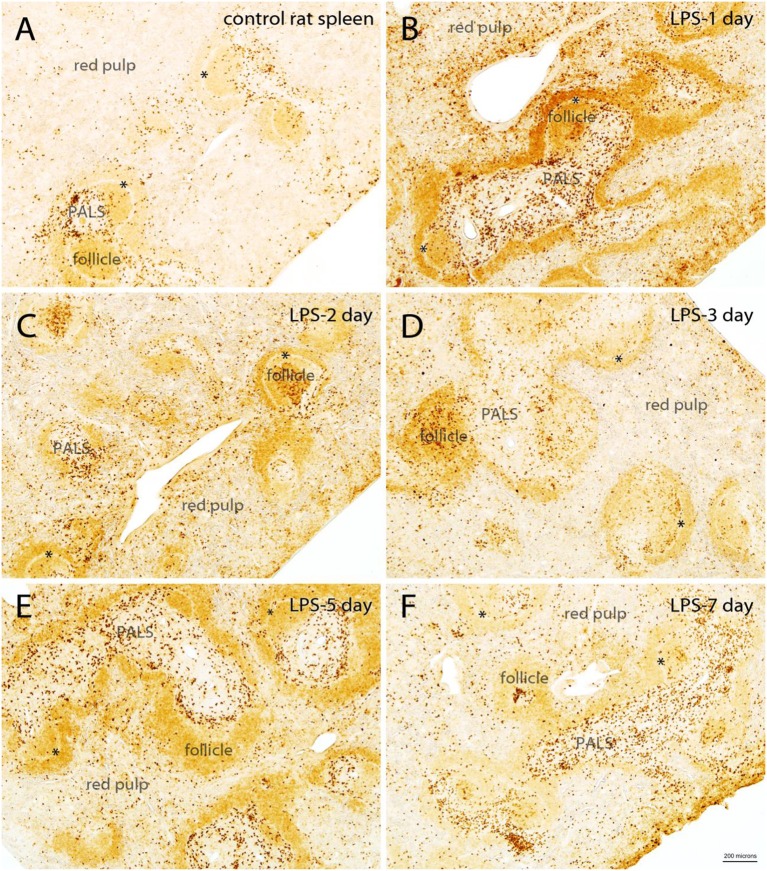
Time course of Quin-IR in the rat spleen after intracerebral injection of LPS into the hippocampus **(A–F)**. Quin-IR was modest in the control rat spleen, but was substantially increased 1 day after injection of 4 μg of LPS into the hippocampus. Strong increases in Quin-IR occurred in all compartments of the spleen (red pulp, PALS, and follicles). Quin-IR remained elevated, but slowly diminished, over the next several days. By day 7, the Quin-IR was returning to near-control levels, but remained elevated. *, marginal sinus. Bar in **(F)**, 200 μm for all images.

Even though we injected LPS only into the hippocampus of rats, a substantial increase in Quin staining was observed in the spleen that persisted for several days following the LPS challenge (Figure 5). In the control spleen from rats, Quin-IR was relatively modest, with very light staining of B-cell follicles, and strong staining of some cells in the T cell PALS (Figure 5A). However, most cells in the PALS were unstained. In the red pulp, which contains macrophages and erythrocytes, scattered macrophages were moderately to strongly stained for Quin in control spleens. In contrast, in the spleen of rats 1 day after injection of 4 μg of LPS into the hippocampus, there was a strong upregulation of Quin-IR in all splenic compartments (Figure 5B). In the PALS, the number of strongly stained cells increased substantially, and staining also increased throughout all B cell follicles. Further, the number of strongly stained macrophages in the red pulp increased as well. On day 2 after LPS administration to the brain, Quin-IR was reduced as compared with day 1, but remained elevated in all three compartments (follicles, PALS, and red pulp; Figure 5C). Strong staining was observed in the central region of some follicles in probable dendritic cells. On day 3 after LPS, Quin-IR remained elevated in all compartments, but was somewhat attenuated as compared with day 2 (Figure 5D). Some follicles continued to harbor strongly stained dendritic cells. B cell follicles remained more immunoreactive than controls. On day 5 after LPS administration, Quin expression in all compartments was again greater than in controls (Figure 5E). Diffuse staining in follicles was elevated and more strongly stained cells were present in the PALS and red pulp, as compared with controls. However, the concentration of strongly stained dendritic cells in the follicles was not as apparent by day 5. By day 7, Quin-IR was beginning to return to pre-LPS levels (Figure 5F). However, the number of stained cells scattered throughout the red pulp and PALS remained elevated relative to controls.

In general, the Quin-IR reaction at the center of follicles, where expression increased in apparent dendritic cells, persisted for at least 3 days. The increase in diffuse staining throughout the B cell-containing follicles lasted for at least 5 days. Elevated numbers of strongly stained cells in the T cell containing PALS, as well as in the red pulp, continued for at least 7 days. These findings indicate a prolonged upregulation of the kynurenine pathway in specific cells of the rat spleen in response to LPS injection into the brain. The major cell types that responded with increased intracellular Quin levels included macrophages, dendritic cells, and B cells. Small, round cells of the T cell-rich PALS were the least immunoreactive for Quin at all-time points. These findings are consistent with macrophages and dendritic cells as major sources of kynurenines and Quin, and that T cells are signaling targets of active tryptophan metabolites, rather than producers.

### Quin-IR in Rat Brain

**Figure 6:**
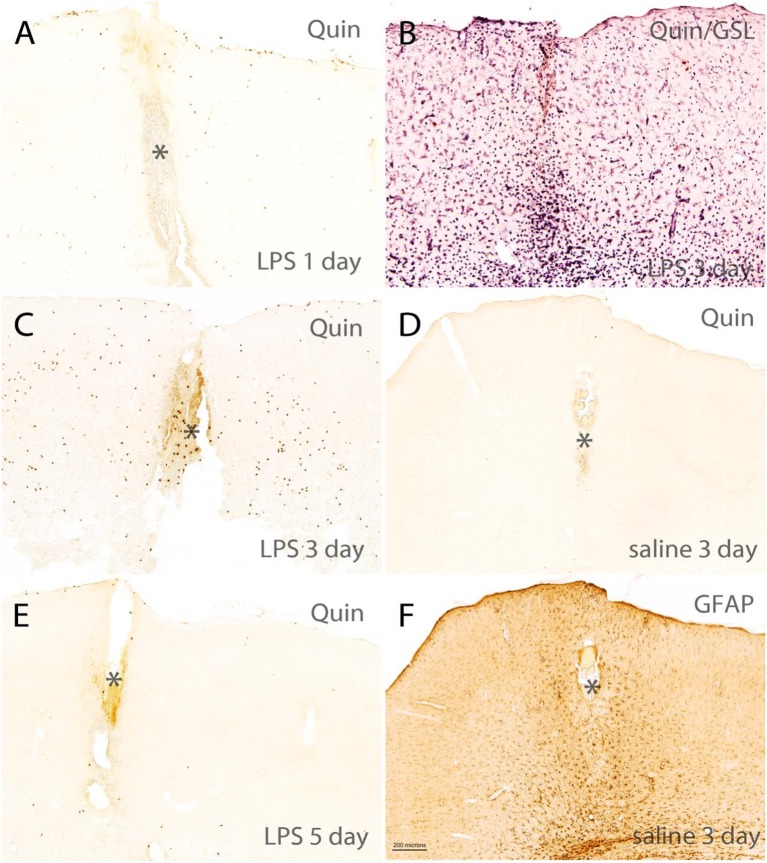
Short-term time course of reactions in rat brain cortex after intracerebral injection of LPS. **(A,C,E)** Show Quin-IR around the injection site in cortex at 1, 3, and 5 days after LPS administration. **(B)** Shows staining for the lectin GSL-IB_4_ (purple chromogen) and Quin (orange chromogen) 3 days after LPS injection, demonstrating the dramatic microglial and macrophage response at 3 days post-injection. **(D)** Shows Quin-IR at the injection site 3 days after saline injection into the hippocampus. **(F)** Shows a moderate astrocyte response (glial acidic fibrillary protein: GFAP) around the injection site 3 days after saline administration, given as control. In the LPS-injected rats, GFAP staining in the damaged area near the injection site was reduced due to astrocyte death. *Injection tract in cortex. Bar in **(F)**, 200 μm in all panels. The orange color in the injection tract is due to hemoglobin in red blood cells.

**Figure 7:**
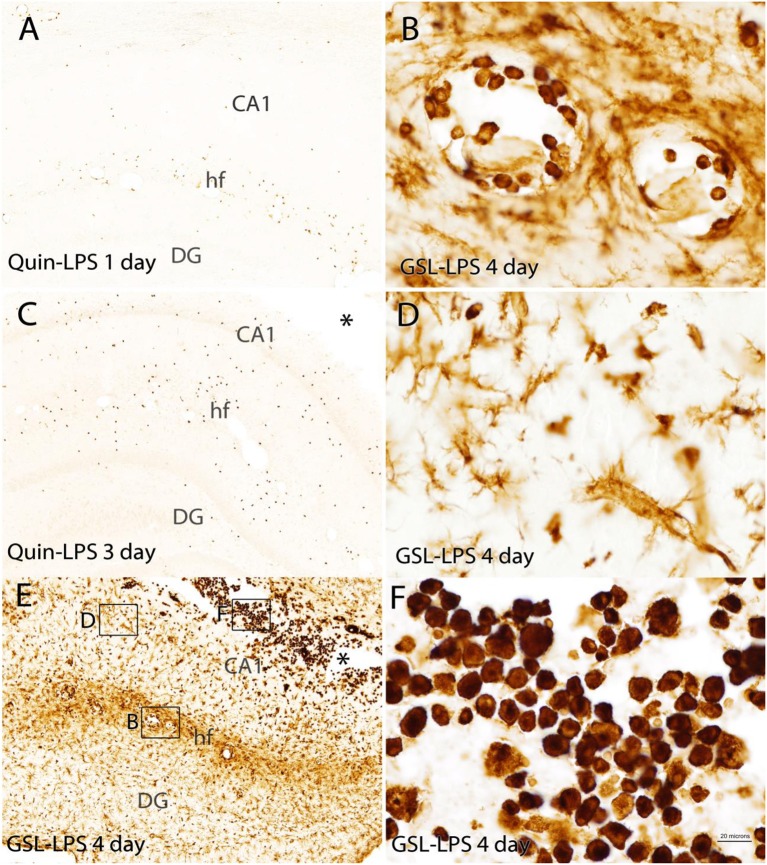
Comparison of the Quin-IR and lectin (GSL) reactions in the hippocampus at several time points after LPS injection. Quin-IR at 1 and 3 days after LPS is shown in **(A,C)**. **(E)** Shows lectin staining for macrophages and activated microglia 4 days after LPS injection. **(B,D,F)** Show enlargements of the lectin staining in areas designated by the boxes in **(E)**. **(B)** Shows monocytes/macrophages adherent to the blood vessels in the hippocampal fissure. **(D)** Shows the strong activation of microglia in the CA1 region of hippocampus. **(F)** Shows the strong infiltration of phagocytes into the necrotic region (*) around the injection site. Bar in **(F)**, 200 μm in **(A,C,E)**; 20 μm in **(B,D,F)**. CA1, CA1 region of hippocampus; DG, dentate gyrus; hf, hippocampal fissure.

A 4-μg dose of LPS was injected into the hippocampus of rats and the brains were fixed with carbodiimide and prepared for immunohistochemistry at various time points. LPS is known to disrupt the blood–brain barrier (41) and injections into the hippocampus elicit strong immune cell infiltration and microglial activation over the course of 7 or more days (42). As expected, high-dose LPS injection into the hippocampus of rats elicited a dramatic, fulminant immune response that resulted in significant local tissue destruction over the course of several days. LPS induced leukocyte recruitment, microglial activation, and astrogliosis. However, a minimal increase of Quin-IR was observed in the brain under these conditions. Twenty-four hours after LPS injection, scattered Quin-IR cells were observed in the meninges, choroid plexus, hippocampal fissure, and in the brain parenchyma in cortex and hippocampus (Figures 6, 7). In the cortex, in the vicinity of the injection track, only scattered Quin-IR cells were present at 24 h (Figure 6A). Three days after LPS administration, the number of Quin-IR cells in the cortex peaked (Figure 6C). Quin-IR cells were observed in the ipsilateral and contralateral hemispheres and extended laterally into temporal cortex on the ipsilateral side. Even so, during this time period, the number of Quin-IR cells in the brain was dramatically exceeded by the number of lectin-stained macrophages and activated microglia (Figures 6B, 7). We also examined animals that had saline injected into the hippocampus as controls. In these animals, a very small number of Quin-IR cells were seen around the injection site on day 3 after the injection (Figure 6D), and astrocytes were reactive as shown by GFAP staining (Figure 6F). By 5 days after LPS injection, the number of Quin-IR in the brain had declined, and scar tissue started to form in the necrotic region around the injection site (Figure 6E).

In the hippocampus, the Quin-IR response was similar to that in cortex (Figure 7). Scattered Quin-IR cells were observed throughout the hippocampus 24 h after LPS (Figure 7A). The maximal Quin-IR response was observed by the third day after LPS injection (Figure 7C). In contrast to Quin expression, the increase in GSL-IB_4_ staining was dramatic in the hippocampus by day 3 (Figure 7E). Lectin-stained cells were adherent to the luminal face of the vasculature (Figure 7B), indicating recruitment of leukocytes from the general circulation. Lectin-stained microglia throughout the hippocampus displayed an activated morphology (Figure 7D). In the necrotic zone around the injection site on day 3, many active phagocytes were strongly stained by GSL-IB_4_ (Figure 7F). The vast majority of these lectin-stained cells were not immunoreactive for Quin (also see **Figure 9**, discussed below).

**Figure 8:**
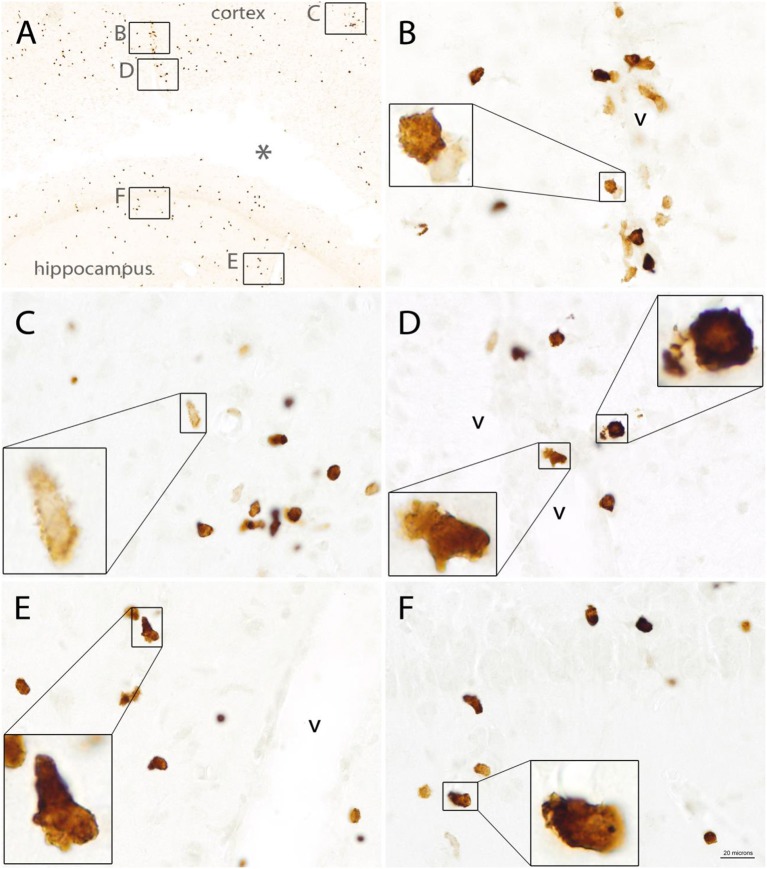
Quin-IR in the vicinity of the injection site 3 days after intracerebral injection of LPS. Quin expression was maximal on day 3 after LPS injection. Immunoreactive cells exhibited highly variable morphology, including lamellipodia and filopodia. Membrane protrusions in the stained cells were very common, indicative of highly mobile cells. Quin-IR cells were scattered around the LPS-affected region **(A)**. Inset areas in **(A)** show magnified regions in **(B**–**F)**. Concentrated Quin-IR cells were observed around the vasculature (v) **(B,D,E,F)**. Some Quin-IR cells were stained at their periphery and had many membrane extensions **(C)**. *Necrotic region around injection site. Bar in **(F)**, 200 μm in **(A)**, 20 μm in **(C–F)**, and 5.5 μm in insets.

**Figure 9:**
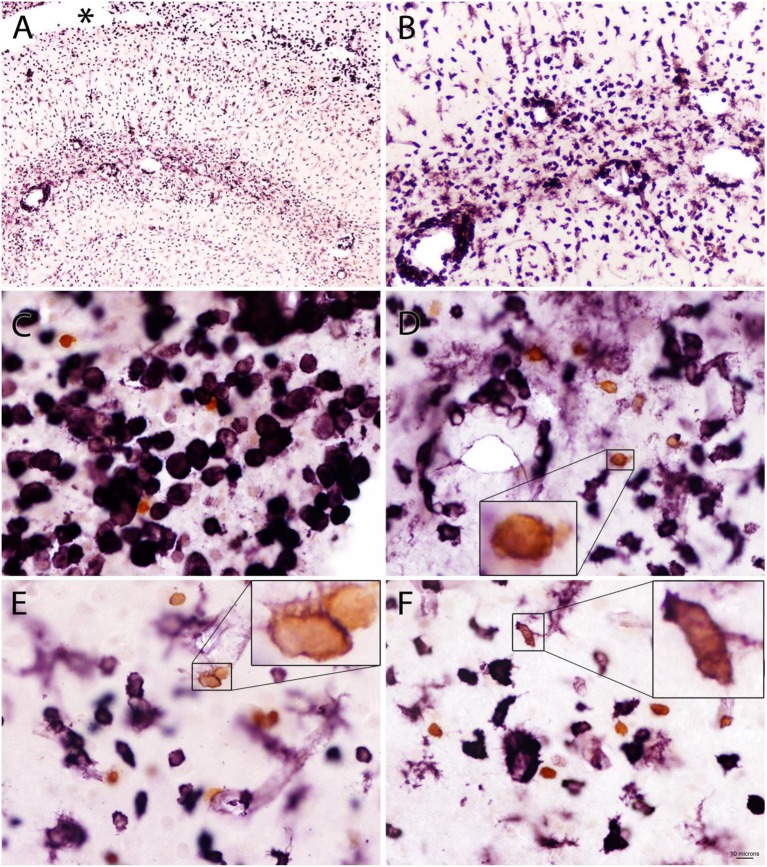
Double staining for Quin-IR (orange chromogen) and GSL-IB_4_ lectin staining (purple chromogen) in the hippocampus 3 days after LPS injection into the hippocampus. Lectin staining showed a massive reaction from phagocytes and microglia surrounding a necrotic region (*) between hippocampus and the overlying corpus callosum **(A)**. Many lectin-stained cells accumulated in and around the vasculature of the hippocampus **(B)**. In contrast to widespread lectin staining of activated microglia and phagocytes throughout the cortex and hippocampus, only a small number of Quin-IR cells was observed **(C–F)**. Some of the Quin-IR cells were stained lightly at their perimeter with the lectin (insets in **D–F**) but many did not have observable lectin staining. Bar in **(F)**, 100 μm in **(A)**, 40 μm in **(B)**, 10 μm in **(C–F)**, and 3 μm in insets.

The morphology of Quin-IR cells was highly variable. Many of the immunoreactive cells in the brain had membrane elaborations including lamellipodia and filopodia (Figure 8). These membrane extensions included knob-like structures (Figure 8B), short, spike-like protrusions (Figure 8C), broad, ruffled extensions (Figure 8D) and various combinations of these (Figures 8E,F). Based on our observations we conclude that most or all Quin-IR cells in the brain after LPS injection are highly motile macrophages. They were generally smaller in diameter than the lectin-stained phagocytic macrophages that occupied the necrotic area (see Figure 9C). Based on their clustering in and around blood vessels in the vicinity of the LPS injection (Figures 8B,D), their apparently motile morphology, as well as their relatively small size, it is very likely that most or all Quin-IR cells observed in the brain were recruited from the peripheral circulation.

Double staining with Quin antibodies and GSL-IB_4_ lectin in the brain after intracerebral LPS injection clearly showed that the strong immune response involving macrophages and reactive microglia was accompanied by relatively restricted Quin accumulation in motile macrophages (Figure 9). GSL-IB_4_ binds to α-galactose residues and is a selective marker for rodent macrophages (43) and microglia (44), and also stained some endothelia. Lectin staining was observed throughout the hippocampus on day 3 after LPS injection (Figure 9A), with many strongly stained cells clustering around the vasculature (Figures 9B,D). Lectin staining was extremely light in control rats, and was mainly seen in some blood vessels (data not shown). Ramified, resting microglia did not stain with the lectin. However, phagocytes (Figure 9C) and reactive microglia stained very strongly with GSL-IB_4_ (Figures 9D–F). After LPS injection, Quin-IR cells were much less frequent than lectin-stained cells, but some Quin-IR cells were also stained at their periphery by GSL-IB_4_ (Figures 9E,F).

**Figure 10:**
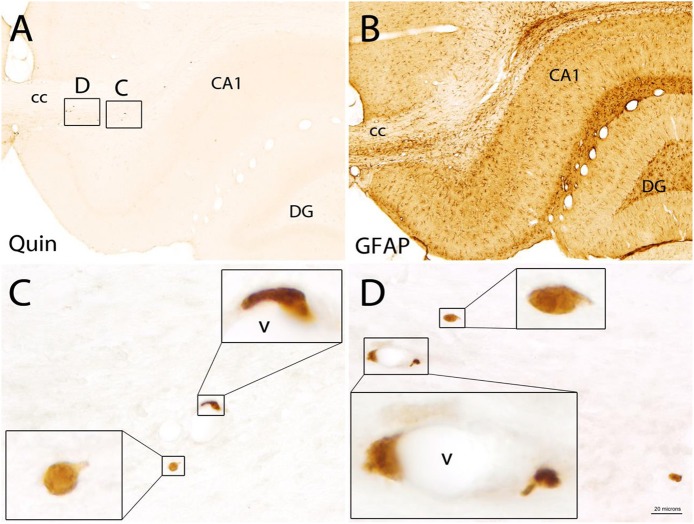
Quin-IR cells in the brain 30 days after LPS injection into the hippocampus. Quin-IR cells were rare near the injection site 30 days after LPS injection **(A)** and were mostly associated with the vasculature (v). GFAP staining in a tissue section adjacent to that in **(A)** is shown in **(B)**. GFAP remained elevated around the injection site at 30 days. The Quin-IR cells exhibited many forms of membrane extension suggestive of diapedesis or migration through tissue **(C,D)**. CA1, area CA1 of hippocampus, cc = corpus callosum, DG = dentate gyrus of hippocampus. Bar in **(D)**, 200 μm in **(A,B)**, 20 μm in **(C,D)**, and 6 μm in panel insets.

Remarkably, some Quin-IR cells were observed within the corpus callosum as late as 30 days after LPS administration (Figure 10). The number of Quin-IR cells was very low (Figure 10A), and GFAP staining remained elevated even at this late time point (Figure 10B). The Quin-IR cells appeared to be infiltrating from the peripheral circulation into the scar area around the injection site (Figures 10C,D). The stained cells again had the morphology of motile macrophages and displayed an array of membrane protrusions and extensions.

We expected to find Quin-IR microglia after direct LPS injection into the brain. This was not apparent, but we did observe some very faintly stained cells that had the morphology of reactive microglia. However, these cells were at the detection limit for immunohistochemistry. Based on the available evidence, microglia do synthesize Quin under these conditions, but they clearly do not accumulate it in their cytoplasm to the same extent as some macrophages.

Based on these findings, we propose that Quin-IR is a useful biomarker for kynurenine pathway activation and metabolite formation up to the level of QPRT and ACMSD (see Figure 1), especially in the periphery. In the brain, the utility of Quin immunohistochemistry as a marker of kynurenine pathway flux is more limited. While it is likely that Quin-IR cells express active kynurenine pathway enzymes from IDO to QPRT, it is possible that not all cells require IDO expression. Our experiments involving tryptophan and kynurenine loading in rats indicate that many cells of the immune system avidly take up kynurenine and convert it to Quin in <3 h (29). This suggests that cells such as fibroblasts that release kynurenine (45) may feed Quin synthesis and NAD^+^ production in other cell types.

### Effect of Kynurenine Administration on Protein Expression in the Mouse Spleen

We investigated the effects of high-dose acute and low-dose prolonged kynurenine loading using two administration methods in mice: IP injection (high-dose acute) and addition to drinking water (low-dose prolonged). For the acute group (*N* = 3 experimental and 3 control), we administered 300 mg/kg of L-kynurenine dissolved in sterile saline, or sterile saline alone, by IP injection 8 h before sacrifice. For the prolonged low-dose administration group, we included 5 mM kynurenine in the drinking water (*N* = 3) as compared with mice given plain water (*N* = 3) for 48 h.

We employed super-physiological doses of kynurenine to study the effects of maximal pathway throughput in the absence of increased TDO or IDO activity. The 300 mg/kg dose is equivalent to 40 mg for a 25-g mouse, which translates to 1.44 mM if distributed completely and uniformly, meaning that the levels in blood and some tissues may have been substantially higher for a short time. The 5 mM dose in drinking water would translate to a dose of approximately 5.2 mg/day/mouse assuming water consumption of 5 ml/day. The use of super-physiological kynurenine doses was done for several reasons. First, we wanted to maximize kynurenine pathway throughput in the absence of immune stimulation or IDO activation. Second, we did not know what concentrations the downstream metabolites would reach in the spleen, especially under conditions where the downstream enzymes had not been activated. Finally, drug dosages for smaller animals often need to be increased relative to larger animals to achieve the same effects, depending on the pharmacodynamics. Nonetheless, we delivered a single IP dose, which would have been mostly cleared from the system during the 8-h post-injection period, and we monitored the proteins that remained altered in expression at 8 h. For the 5 mM dose in drinking water, the administration was intermittent during waking hours, and therefore would have remained elevated throughout that period.

One noteworthy feature of kynurenine loading is production of kynurenic acid, as the relevant kynurenine aminotransferase reaction (see Figure 1) is dependent on the high substrate concentrations due to the high Km of the enzyme for kynurenine (46). For example, intraperitoneal administration of kynurenine induces dose-dependent increases in rat brain cortical and striatal kynurenic acid concentrations and a dramatic elevation of its extracellular striatal levels (47, 48). As such, kynurenine loading with high doses would lead to saturation of pathway enzymes and increase kynurenic acid formation by kynurenine aminotransferase. Kynurenic acid may be one of the more important ligands for activating the aryl hydrocarbon receptor (AHR; discussed below).

### 2D Gel Analysis

The quantitative fluorescent 2D-DIGE proteomic methods have been described previously (33) and involve labeling protein samples from control and experimental groups with distinct fluorescent dyes prior to 2D gel analysis (49). The experimental and control samples are then mixed and run together on 2D gels along with an internal standard. Only protein spots that were altered in ratio in the same direction between all three experimental and control animals were considered. This system has been found to provide sensitive and reproducible results with low protein samples (50). In the current study, over 1,000 protein spots were resolved on the 2D gels at both time points (Supplementary Figure 2). In all, 115 protein spots were altered at the two time points that had significantly changed ratios (*p* < 0.05) between control and experimental groups, and among these, 92 proteins were identified by mass spectroscopy.

### Mass Spectroscopy; Acute (8 h) Group

In mice given a single 300 mg/kg injection of L-kynurenine 8 h before sacrifice, a total of 69 protein spots were found to change in abundance relative to controls. Among these, 55 changes were statistically significant (*p* < 0.05), and 41 of the proteins were identified by mass spectroscopy (Table 1). Fourteen of the protein spots that had statistically significant variable ratios between experimental and control groups were not identified by mass spectroscopy.

**Table 1:** Identified protein spots from spleen that were significantly increased or decreased 8 h after IP kynurenine administration (300 mg/kg).

**Protein name**	**Protein ID**	**Av. ratio**	***T*-test**
Actin, cytoplasmic 1 (Beta-actin)	ACTB_HUMAN	1.92	0.037
Actin, cytoplasmic 1 (Beta-actin)	P02570	1.62	0.025
Endoplasmin precursor	ENPL_MOUSE	1.43	0.003
Vascular cell adhesion protein 1 precursor (V-CAM1)	VCA1_MOUSE	1.42	0.00084
Adipocyte-derived leucine aminopeptidase precursor (ERAP1)	ART1_MOUSE	1.4	0.013
Coronin-like protein p57 (Coronin 1A)	CO1A_MOUSE	1.37	0.0072
Alpha-actinin 4 (Non-muscle alpha-actin 4)	P57780	1.35	0.019
Gelsolin precursor, plasma (Actin-depolymerizing factor)	GELS_MOUSE	1.35	0.048
Actin, cytoplasmic 1 (Beta-actin)	ACTB_HUMAN	1.33	0.018
Actin, cytoplasmic 1 (Beta-actin)	ACTB_HUMAN	1.33	0.017
Calreticulin precursor (CRP55) (Calregulin) (HACBP) (ERp60)	P14211	1.37	0.0033
SAM domain and HD domain-containing protein 1	SAD1_MOUSE	1.33	0.02
Actin, cytoplasmic 1 (Beta-actin)	ACTB_CRIGR	1.31	0.0027
Calreticulin precursor (CRP55) (Calregulin) (HACBP) (ERp60)	P14211	1.31	0.0027
WD-repeat protein 1 (Actin interacting protein 1)	WDR1_MOUSE	1.3	0.0041
Transitional endoplasmic reticulum ATPase (TER ATPase)	P46462	1.3	0.019
Gelsolin precursor, plasma (Actin-depolymerizing factor)	P13020	1.27	0.028
Elongation factor 2 (EF-2)	EF2_MOUSE	1.27	0.025
Actin, cytoplasmic 1 (Beta-actin)	P02570	1.24	0.00076
Moesin (Membrane-organizing extension spike protein)	P26041	1.24	0.0089
Heat shock protein HSP 90-alpha (HSP 86)	HS9A_MOUSE	1.24	0.024
Heat shock protein HSP 90-alpha (HSP 86)	P07901	1.24	0.018
Gelsolin precursor, plasma (Actin-depolymerizing factor)	GELS_MOUSE	1.23	0.0031
Peroxiredoxin 2 (EC 1.11.1.-) (Thioredoxin peroxidase 1)	Q61171	1.23	0.019
Pyruvate dehydrogenase E1 component beta subunit	ODPB_RAT	1.2	0.0032
Radixin	RADI_MOUSE	1.18	0.0031
Sorting nexin 5	Q9D8U8	−1.1	0.017
Tubulin beta-5 chain	P05218	−1.11	0.014
Glyceraldehyde 3-phosphate dehydrogenase (EC 1.2.1.12)	P16858	−1.14	0.0037
L-lactate dehydrogenase A chain (EC 1.1.1.27) (LDH-A)	P06151	−1.14	0.0043
Heterogeneous nuclear ribonucleoproteins A2/B1	O88569	−1.15	0.0066
Alpha enolase (EC 4.2.1.11)	ENOA_MOUSE	−1.15	0.015
Tubulin alpha-3/alpha-7 chain (Alpha-tubulin 3/7)	TBA3_MOUSE	−1.16	0.0071
Heterogeneous nuclear ribonucleoproteins A2/B1	O88569	−1.17	0.013
Tubulin alpha-1 chain	TBA1_MOUSE	−1.18	0.011
Hsp90 co-chaperone Cdc37 (Hsp90 chaperone protein)	CC37_MOUSE	−1.21	0.024
Adenylyl cyclase-associated protein 1 (CAP 1)	P40124	−1.23	0.02
Coronin 1B (Coronin 2)	CO1B_MOUSE	−1.26	0.035
Serum albumin precursor	P07724	−1.34	0.043
Heterogeneous nuclear ribonucleoprotein K	ROK_HUMAN	−1.36	0.044

*All identified spots that were changed significantly are shown, including multiple spots with the same protein*.

Ten cytoskeletal protein spots were changed in abundance, six were expressed at higher levels, and four were reduced. Beta-actin was identified in six distinct spots on the gels, and the expression of this isoform of actin was increased in all six spots, with the experimental to control ratios ranging between +1.23 and +1.92. The mass fingerprint for gelsolin was found in three distinct spots on 2D gels, and all were increased relative to controls (average ratios of +1.23, +1.27 and +1.35). Two distinct protein spots representing radixin and moesin were both increased in the spleens of animals 8 h after kynurenine administration (ratios of +1.18 and +1.24, respectively). Actin interacting protein 1 (also called WD repeat protein 1) was increased in abundance, with a ratio of +1.3, and coronin 1a was also increased, with a ratio of +1.37. In contrast, coronin 1b was decreased in abundance at 8 h, with a ratio of −1.26. Three isoforms of tubulin had decreased ratios, including tubulin beta-5 (−1.11), tubulin alpha3/alpha7 (−1.16), and tubulin alpha1 (−1.18). VCAM-1, a cell adhesion molecule that is involved in leukocyte adhesion and diapedesis, was increased 8 h after kynurenine administration (ratio of +1.42).

At the 8-h time point, several protein expression changes were notable. These included eukaryotic translation elongation factor 2 (EF-2, also EEF-2) (ratio +1.27), which positively regulates translation of certain proteins including heat shock protein 90 (HSP90) (51) and TNF-α (52). Also, valosin-containing protein (VCP, also known as transitional endoplasmic reticulum ATPase or p97) had an increased ratio of +1.30. VCP is involved in several processes including protein quality control (ERAD), autophagy, and autophagosome–lysosome fusion, among others (53). Interestingly, heat shock protein 90B1 (HSP90B1; also known as endoplasmin), which is associated with the HSP90 chaperone complex, was expressed at an increased ratio +1.43. Sorting nexin 5 (SNX5), which is involved in macropinocytosis and antigen processing, had a reduced ratio of −1.10 in the 8-h acute kynurenine group as compared with controls.

Two immune cell-specific proteins were upregulated at 8 h, including endoplasmic reticulum aminopeptidase 1 (ERAP1; increased average ratio of +1.40) and SAM domain- and HD domain-containing protein 1 (ratio of +1.33). Mice lacking ERAP1 had reduced numbers of both Tr1-like regulatory T cells and tolerogenic dendritic cells (54), suggesting that increased ERAD1 expression could be associated with increased numbers of these tolerogenic cell types in the spleen in response to kynurenine administration. SAM and HD domain-containing protein 1 (SAMHD1) is involved in anti-viral responses (55).

Two ribonucleoproteins that have been associated with apoptosis were downregulated at 8 h, including heterogeneous nuclear ribonucleoproteins A2/B1 (reduced ratios of −1.17 and −1.15 in two spots) and heterogeneous nuclear ribonucleoprotein K (hnRNPK: reduced ratio of −1.36). hnRNPK is a DNA-binding protein that can act as a transcription enhancer or repressor (56).

One antioxidant enzyme, peroxyredoxin 2, was changed in abundance 8 h after kynurenine administration (ratio of +1.23). Additionally, three chaperone proteins were differentially expressed in experimental animals relative to controls, including heat shock protein 90-alpha (HSP90-alpha; ratios of +1.24 in two distinct spots), calreticulin (calregulin; increased ratios of +1.37 and +1.31 in two spots), and HSP90 co-chaperone (CDC37, reduced ratio of −1.21).

### Mass Spectroscopy; Prolonged (48 h) Group

In mice administered 5 mM kynurenine in their drinking water for 48 h, 60 protein spots were differentially expressed at significant levels (*p* < 0.05) of which 51 were identified by mass spectroscopy (Table 2). Nine additional spots that were significantly altered in expression could not be identified.

**Table 2:** Protein expression changes in mouse spleen after mice were exposed to 5 mM kynurenine in drinking water for 48 h.

**Protein name**	**Protein ID**	**Av. ratio**	***T*-test**
Moesin	MOES_MOUSE	1.88	0.00099
Gelsolin precursor, plasma	GELS_MOUSE	1.77	0.0067
Elongation factor 2 (EF-2)	EF2_RAT	1.69	0.00034
Gelsolin precursor, plasma	GELS_MOUSE	1.68	0.026
SAM domain and HD domain-containing protein	SAD1_MOUSE	1.62	0.0067
C-1-tetrahydrofolate synthase, cytoplasmic	C1TC_RAT	1.62	0.015
DNA replication licensing factor MCM7	MCM7_MOUSE	1.5	0.0095
Gelsolin precursor, plasma	GELS_MOUSE	1.5	0.013
Gelsolin precursor, plasma	GELS_MOUSE	1.49	0.029
Non-muscle caldesmon (CDM) (L-caldesmon)	CALD_RAT	1.48	0.015
Moesin	MOES_MOUSE	1.47	0.036
Ezrin (p81) (Cytovillin) (Villin 2)	EZRI_MOUSE	1.43	0.0064
Radixin (ESP10)	RADI_MOUSE	1.43	0.039
Radixin (ESP10)	RADI_MOUSE	1.4	0.024
Phosphatidylinositol 3-kinase regulator	P85A_MOUSE	1.38	0.00013
Endoplasmin precursor	ENPL_MOUSE	1.35	0.0077
Transketolase (EC 2.2.1.1) (TK) (P68)	TKT_MOUSE	1.33	0.012
Transketolase (EC 2.2.1.1) (TK) (P68)	TKT_RAT	1.33	0.031
Elongation factor 2 (EF-2)	EF2_MOUSE	1.33	0.017
Vacuolar protein sorting 35	VP35_MOUSE	1.32	0.019
Gelsolin precursor, plasma	GELS_MOUSE	1.31	0.027
Transketolase (EC 2.2.1.1) (TK) (P68)	TKT_MOUSE	1.31	0.0042
Osmotic stress protein 94	OS94_MOUSE	1.31	0.028
Keratin, type I cytoskeletal 10	K1CJ_MOUSE	1.29	0.017
Elongation factor 2 (EF-2)	EF2_MOUSE	1.29	0.015
Endoplasmin precursor	ENPL_MOUSE	1.28	0.0057
Heat shock 70-related protein APG-2	HS74_MOUSE	1.28	0.00048
Heat shock protein HSP 90-beta (HSP 84)	HS9B_RAT	1.26	0.0061
Alpha-actinin 4	AAC4_MOUSE	1.24	0.034
Elongation factor 2 (EF-2)	EF2_MOUSE	1.23	0.014
Structure-specific recognition protein 1	SSRP_MOUSE	1.22	0.044
Hematopoietic lineage cell specific protein	HS1_MOUSE	1.22	0.0075
Serum albumin	ALBU_RAT	1.22	0.033
Endoplasmin precursor	ENPL_MOUSE	1.21	0.035
14-3-3 protein epsilon	143E_HUMAN	1.06	0.02
Swiprosin 1	SWS1_MOUSE	−1.14	0.04
26S proteasome non-ATPase reg. subunit	PSDD_MOUSE	−1.14	0.0084
Keratin, type I cytoskeletal 10	K1CJ_MOUSE	−1.16	0.018
T-complex protein 1, theta subunit	TCPQ_MOUSE	−1.19	0.0098
Tubulin alpha-1 chain	TBA1_MOUSE	−1.19	0.016
Activator of 90 kDa heat shock protein	AHA1_MOUSE	−1.21	0.037
Actin, cytoplasmic 1 (Beta-actin)	ACTB_CRIGR	−1.21	0.014
Actin, cytoplasmic 1 (Beta-actin)	ACTB_CRIGR	−1.21	0.003
Thioredoxin domain containing protein 4	TXN4_MOUSE	−1.22	0.0051
Thioredoxin domain containing protein 4	TXN4_MOUSE	−1.25	0.011
Hsc70-interacting protein (Hip)	ST13_MOUSE	−1.27	0.023
60 kDa heat shock protein	CH60_MOUSE	−1.36	0.015
Heterogeneous nuclear ribonucleoprotein	ROK_HUMAN	−1.38	0.027
Actin, cytoplasmic 1 (Beta-actin)	ACTB_CRIGR	−1.97	0.0037
Ubiquitous tropomodulin (U-Tmod)	TMO3_MOUSE	−1.99	0.0095
14-3-3 protein zeta/delta (YWHAZ)	143Z_MOUSE	−2.31	0.0035

*All identified proteins that were changed significantly are included, showing identification of multiple spots representing the same protein*.

The suite of cytoskeletal protein changes at 48 h was somewhat different from that observed at 8 h. Ten cytoskeletal proteins were changed in abundance in the kynurenine group, with seven showing increased expression, and three having decreased expression. Gelsolin was upregulated in five distinct protein spots, with increased ratios ranging from +1.31 to +1.77. The important cytoskeletal regulatory proteins ezrin, radixin, and moesin were all upregulated in experimental animals, and both radixin and moesin were present in two distinct spots on gels. Alpha-actinin-4 showed an increased ratio of +1.24, and non-muscle caldesmon had an increased ratio of +1.48. There were four cytoskeletal proteins that were reduced in abundance 48 h after kynurenine administration, including tubulin alpha-1 chain (ratio reduced by −1.19), keratin type 1 (1 spot with a positive ratio of +1.29, and 1 spot with a negative ratio of −1.16), ubiquitous tropomodulin (negative ratio of −1.99), and beta-actin (3 spots with negative ratios of −1.97, −1.21, and −1.21).

Several proteins associated with apoptosis showed differential expression at 48 h, including 14-3-3 zeta/delta, which had a ratio of −2.31 (a >50% drop). This was the largest single change observed in the study. Also, 26S proteasome non-ATPase regulatory subunit 13 was slightly decreased in abundance (ratio −1.14). A number of other proteins that were altered 8 h after kynurenine administration have connections to the regulation of apoptosis. For example, ezrin is involved in apoptotic regulation by linking the actin cytoskeleton to CD95. Heterogeneous nuclear ribonuclear protein K has also been associated with apoptosis (57). Elongation factor 2 inhibition has been associated with apoptosis, but in the present study, we see increases in abundance of this protein (58).

Three immune system associated proteins were increased in the spleens of experimental animals at 48 h. SAM domain- and HD domain-containing protein, which was also elevated at 8 h, was further increased in abundance at 48 h (ratio +1.62). Hematopoietic lineage cell specific protein 1 (HS1) was increased (ratio +1.22), as was structure-specific recognition protein 1 (SSRP-1; ratio +1.22). HS1 induces actin polymerization and branching in hematopoietic cells by interacting with the ARP2/3 complex (59). HS1 is also a caspase target during apoptosis (60). SSRP-1 can act as either a transcriptional activator, or co-activator, and plays multiple roles in the regulation of transcription. However, it has been shown that overexpression of SSRP1 with p63-gamma caused a ~43% increase of apoptotic cells (61).

Several growth or protein translation/translocation associated proteins were increased in expression after 48 h of kynurenine administration. Elongation factor 2 (EF-2, also EEF-2), which is associated with protein chain elongation on ribosomes, was found at four spots on gels, and these had increased ratios of +1.23, +1.29, +1.33, and +1.69. Transketolase was present in three distinct spots on the gels, all of which showed increased ratios at 48 h (ratios of +1.33, +1.33, and +1.31). DNA replication licensing factor MCM7, which is associated with controlling cell cycle check points, was increased in abundance (ratio +1.5). Vacuolar protein sorting 35 was upregulated at 48 h (ratio +1.32).

Seven chaperone or heat shock-related proteins were differentially expressed after 48 h of kynurenine administration (3 increased, 4 decreased). Heat shock protein 90-beta (HSP90-beta) was increased (ratio +1.26), as were osmotic stress protein 94 (ratio +1.31) and heat shock 70-related protein APG-2 (ratio +1.28). The four proteins in this category that were decreased in abundance included T-complex protein 1-theta (ratio −1.19), heat shock protein 60 (HSP60, ratio −1.36), activator of 90 kDa heat shock protein (AHSA1, also AHA1; ratio −1.21), thioredoxin domain-containing protein 4 (two spots: ratios −1.22 and −1.25), and HSC70-interacting protein (ST13, also HIP; ratio −1.27). High expression levels of ST13 are associated with reduced cell migration and proliferation of colorectal cancer cells (62). Swiprosin 1, which is involved in cortical cytoskeleton dynamics among other functions (63, 64), was also decreased in the spleen after 48 h of kynurenine administration (ratio −1.14).

**Figure 11:**
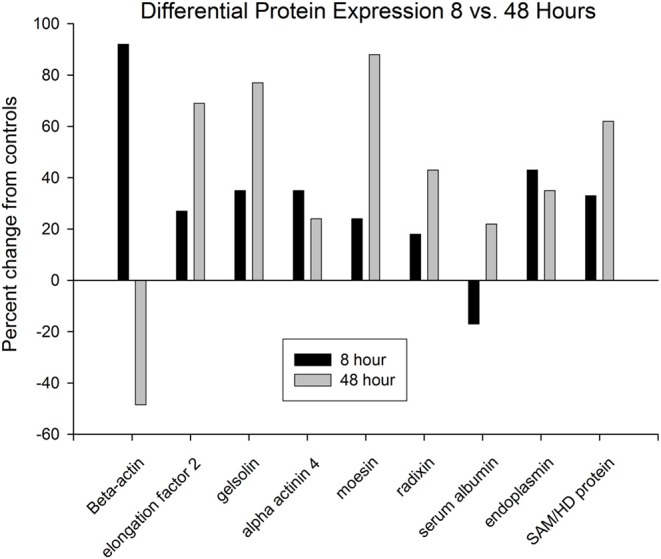
Only nine proteins were identified that were differentially expressed at both time points. Seven of these increased at both time points, whereas two proteins displayed reversed expression patterns at the two time points. Beta-actin was increased at 8 h, but decreased at 48 h, whereas serum albumin was decreased at 8 h, but increased at 48 h.

Only nine of the identified proteins that had altered ratios at 8 h were also changed at 48 h (Figure 11). Among these, seven of the protein expression changes at 8 and 48 h were in the same direction (upregulated). In contrast, two proteins, beta-actin and serum albumin, reversed expression levels between the two time points. Because both serum tryptophan and kynurenine are substantially albumin-bound (65), the observed changes in serum albumin levels would be expected to alter the free/bound tryptophan and kynurenine ratios, and downstream flux through the kynurenine pathway. At both time points, several proteins were observed in multiple positions on the gels. This was particularly true of the 48-h group, in which 16 identified proteins were present at two or more spots. This could have been due to increased apoptosis and caspase-mediated proteolysis, and/or due to post-translational modifications. Many of the protein expression changes observed after kynurenine administration were associated with the dynamic actin cytoskeleton, or with cell migration, proliferation or apoptosis.

## Discussion

The brain is an immune-privileged organ, with heightened barriers to peripheral immune cell trafficking, and therefore, it does not respond to immune challenges to the same degree as many peripheral organ systems [reviewed in (66)]. This was clearly observed with Quin immunohistochemistry in our models involving peripheral and central immune stimulation. Because the brain is less responsive to certain types of immune stimulation as compared with peripheral organ systems, there may be less need for the immunosuppressive kynurenine metabolites in the brain. Even though kynurenine pathway activity in the brain is lower than in the periphery, the kynurenine pathway has been shown to be critical for immune privilege in the brain (67). Limited Quin production in the brain remains a poorly understood aspect of the immune-privileged status. Microglia are fully capable of generating Quin if provided super-physiological levels of the immediate precursor (31), but we observed little or no Quin-IR in microglia after direct injection of LPS into the hippocampus. The LPS injection needles breached the meningeal and blood–brain barriers, resulting in a strong immune response that activated microglia and recruited peripheral immune cells. Most of the scattered Quin-IR cells in the brain after LPS injection appeared to be infiltrating monocytes/macrophages. The immunosuppressive effects of kynurenine pathway metabolites are important mechanisms employed by the immune system to prevent excess responses that harm the host, at least outside of the brain. But due to its immune privileged status, and the neurotoxic nature of Quin, the brain does not appear to task the kynurenine pathway with preventing excess activation of immune responses to the same extent as in the periphery. Instead, we propose that the brain channels kynurenine pathway metabolism to the synthesis of metabolites other than Quin, and the Quin that is generated is rapidly converted to NAD^+^, rather than accumulated in the cytoplasm of cells.

In contrast to Quin immunohistochemistry, lectin staining showed a dramatic immune response in the brain after LPS injection involving microglia and infiltrating macrophages. The immune reaction to LPS was accompanied by extensive tissue damage to the hippocampus, corpus callosum, and cortex that was maximal at 3 days after LPS injection. The number of Quin-IR cells in the brain represented a very small fraction of the observed macrophages and activated microglia as shown by lectin histochemistry (see Figures 6, 7, 9). This clearly indicates that the vast majority of the macrophages, microglia, and other cells that responded to the LPS challenge were not immunoreactive for Quin. These results do not support a major role for excessive Quin formation as causative in the tissue damage that occurred in response to LPS injection into the hippocampus.

The Quin-IR response in the liver and spleen of gerbils after systemic PWM administration was dramatic (Figures 3, 4). Gerbils lack the apoenzyme form of tryptophan dioxygenase and therefore cortisol does not regulate tryptophan catabolism in the liver as it does in certain other species, including rats and humans (68). Thus, tryptophan catabolism in the gerbil liver is constitutive and systemic tryptophan levels are generally lower than in many other species. In the gerbil spleen, the red pulp transitioned from virtually no Quin-IR in the saline-treated animals to a strong and nearly ubiquitous distribution 24 h after PWM administration. In the white pulp, the PALS dramatically increased Quin expression, whereas staining in the B cell-containing follicles increased modestly. Based on morphology and location within the compartments of the spleen, the majority of Quin-IR cells were macrophages and dendritic cells. Much lower immunoreactivity was observed in areas rich in T and B cells.

In the brain of gerbils after peripheral PWM stimulation, Quin-IR cells were only observed in the choroid plexus, vasculature, and meninges. Some Quin-IR cells in the choroid plexus had the appearance of foam cells, and were filled with large intracellular inclusions (Figures 2B,E). Foam cells are lipid-laden macrophages that have taken up oxidized lipids, and this form of macrophage has been associated with disorders including atherosclerosis (69, 70). Similarly, in the liver of gerbils after PWM administration, some Kupffer cells were filled with many large intracellular inclusions that could represent phagolysosomes (Figure 4F). The kynurenine metabolite 3-hydroxyanthranilate has been found to affect foam cell formation by reducing uptake of oxidized low-density lipoprotein by macrophages (71, 72). These findings suggest that kynurenine pathway metabolites may be linked to phagocytosis.

The morphology of Quin-IR cells was varied in different tissues. Some cells in the follicles and PALS of the gerbil spleen after PWM administration had the appearance of dendritic cells (Figure 3F). In the rat spleen after LPS injection into the hippocampus, many Quin-IR cells could be identified as macrophages and dendritic cells based on location and morphology. In the rat brain after LPS injections, the cells tended to be relatively small, and often had membrane elaborations including lamellipodia and filopodia (Figures 8, 9). Overall, Quin-IR cells in the brain after LPS injection resembled motile macrophages [see (73)]. Many Quin-IR cells had multiple knob-like membrane protrusions that have been referred to as “blebs.” We also observed Quin-IR cells with filopodia and lamellipodia. Motile macrophages employ the cortical cytoskeleton to generate membrane protrusions that facilitate cell migration through tissues [reviewed in (74)]. We observed a wide array of membrane extensions in Quin-IR cells after LPS injection into the brain.

In our 2D-DIGE proteomics investigation, kynurenine pathway flux was engaged in the absence of immune stimulation, IDO induction, tryptophan depletion, or interferon-gamma administration. The significant spleen protein changes observed resulted from exposure to kynurenine pathway metabolites alone, further confirming that these molecules have modulatory and regulatory roles in leukocyte protein expression and function.

Selective induction of apoptosis is a frequently cited action of some kynurenine metabolites (75). In the current proteomic study, six protein spots that were significantly increased at 8 h and seven spots increased at 48 h were found to have the sequence of beta actin. These distinct spots containing related amino acid sequences might suggest that several post-translationally modified forms of actin were formed in response to kynurenine administration. However, cytoskeletal proteins are also important targets for caspase-mediated proteolysis during apoptosis, and the presence of beta-actin in multiple locations on gels could be due to proteolytic breakdown. A notable finding of the current study was that numerous protein targets of kynurenine catabolite action in the spleen were cytoskeletal including ERM proteins (ezrin, radixin, and moesin), gelsolin, WD repeat protein-1, and coronin-like protein p57, as well as actin and tubulin isoforms. A plausible explanation of these protein changes could be that kynurenine catabolites induce selective leukocyte apoptosis, because many of these cytoskeletal proteins are primary targets for caspase-3-mediated proteolysis (60, 76). However, these findings could also suggest that kynurenines may play a role in regulating the dynamics of the actin cytoskeleton in leukocytes based on the levels of kynurenine metabolites.

In leukocytes, ERM proteins are involved in cell polarization, extension of lamellipodia and filopodia, chemotaxis, formation of the immunological synapse, intracellular granule movement, and phagocytosis. ERM protein action is regulated by a system of kinases and phosphatases that control the level of phosphorylation in response to various signals. Phosphorylation is associated with ERM activation, association of ERM with the cortical actin cytoskeleton, and the formation of cellular microvilli. Proteomic data in the present study showed that ERM proteins were significantly increased at 48 h, and both moesin and radixin were present in two distinct protein spots with increased ratios. ERM proteins are critically involved in the formation of the immunological synapse between T cells and antigen-presenting cells (77), and their phosphorylation is associated with the exclusion of certain surface proteins from the contact zone between the two cell types. The finding that endoplasmic reticulum aminopeptidase I (gene ERAP1) was increased in abundance after kynurenine administration may relate to antigen presentation by APCs during the formation of the immunological synapse. Interestingly, the NAD^+^-dependent deacetylase enzyme SIRT1 is required for lamellipodia extension and migration of melanoma cells (78). Sirtuins including SIRT1 consume NAD^+^ and therefore it is possible that migrating cells benefit from an increased supply. This raises the possibility that local supply of NAD^+^ at the leading edge of migrating cells could be generated from stored intracellular Quin if QPRT, NMNAT, and NADSYN1 are also expressed locally (see Figure 1).

Links between the kynurenines and cell motility come from research into the AHR and cancer. The AHR is a transcription factor that responds to a number of different signaling agents including several of the kynurenine pathway metabolites. In particular, kynurenine, kynurenic acid, and xanthurenic acid have been shown to activate the AHR (79–82). The AHR is the primary receptor system that kynurenine metabolites act through. Inactive AHR is retained in the cytoplasm bound to HSP90, and ligand binding dissociates AHR from HSP90 and exposes a nuclear localization signal. AHR is then transported to the nucleus where it forms a transcription factor complex and binds to specific DNA response elements [reviewed in (83)]. In the current study, we found a number of changes in HSP90-related protein levels [HSP90 reviewed in (84)]. HSP90-beta (HSP90AA1; HSP86) had an increased ratio of 1.24-fold 8 h after kynurenine administration, whereas HSP90 co-chaperone (CDC37) was decreased by −1.21-fold at this time point. HSP90-alpha (HSP90AB1; also known as HSP84) had an increased ratio of +1.26 after 48 h of kynurenine administration in drinking water. Also at 48 h, activator of 90 kDa heat shock protein (AHSA1, also AHA1) was decreased −1.21-fold. AHSA1 has been reported to regulate proliferation, apoptosis, migration, and invasion of osteosarcoma (85). Silencing of AHSA1 via siRNA significantly reduced migration of osteosarcoma cells in culture. Finally, at both time points, endoplasmin precursor (HSP90B1) was upregulated (see Figure 11). HSP90B1 overexpression has been associated with metastasis of breast cancer (86). Our results on the upregulation of HSP90 protein expression after kynurenine administration are consistent with the conclusion that the AHR/HSP90 system acts to regulate responses to kynurenine metabolites.

There are a number of published reports that indicate the AHR is somehow linked to cell motility and migration. For example, in human breast cancer cell lines, Novikov et al. found that a combination of 50 μM kynurenine and 10 μM xanthurenic acid exerted a maximal effect on promoting cell migration (80). This effect was mediated by the AHR system, but in the case of breast cancer cells, AHR activation was linked to increased TDO activity, rather than IDO. Activation of the AHR in epithelial cells led to morphological changes associated with cytoskeletal remodeling, increased interaction with the extracellular matrix, and reduced cell–cell contacts (87). AHR activation in lung fibroblasts significantly enhanced migration via generation of arachidonic acid metabolites (88). AHR agonists also significantly increased migration of squamous cell carcinoma cells in culture (89). Our data are consistent with these findings and suggest that kynurenine metabolites lead to changes in the expression levels of HSP90-related proteins that impact on cell motility and other cellular behaviors. We propose that kynurenine and its derivatives facilitate cell motility and cancer metastasis via activation of the AHR/HSP90 system, resulting in dissociation of the cytoplasmic complex and nuclear translocation of AHR, which activates genes that are involved in enhanced cytoskeletal dynamics and increased cell motility.

### Hepatic vs. Immune Kynurenine Pathways and the Kynurenine Switch

Not all enzymes of the kynurenine pathway are present in all cells, so the complement of expressed enzymes is key to how tryptophan is catabolized in a particular cell type (see Figure 1). Hepatocytes express the full repertoire of kynurenine pathway enzymes including the NAD^+^ synthetic and oxidation pathways. As such, the liver is the primary site of tryptophan metabolism under normal physiological conditions. The regulation of hepatic TDO and non-hepatic IDO kynurenine pathway activation is distinct. An important activator of TDO is cortisol, whereas interferon-γ is one of the more potent inducers of IDO activity (90, 91).

**Figure 12:**
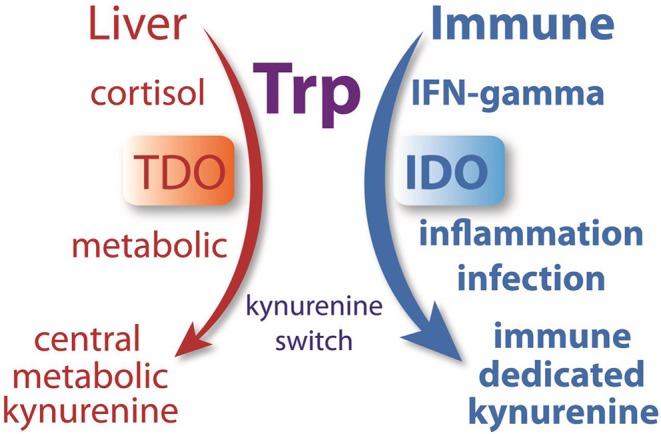
Many researchers have hypothesized that the strong induction of IDO during infection was directed at reducing tryptophan levels in order to control pathogen replication or induce tolerance. We hypothesize that IDO, which is upregulated in many tissues by immune-activating agents such as INF-γ, initiates a system-wide diversion of tryptophan metabolism from the liver to the immune system. During normal metabolism, cortisol activates TDO in hepatocytes to metabolize tryptophan. During an immune response, INF-γ activates IDO in many cell types, converting tryptophan to kynurenine, which is then released to the circulation where it can be preferentially taken up by cells of the immune system. This enzyme-mediated kynurenine switch diverts tryptophan metabolism and NAD^+^ synthesis from the liver to the immune system during an immune response.

In hepatocytes, any tryptophan that is not required for protein synthesis can be converted to NAD^+^ or oxidized internally to CO_2_. As such, most systemic tryptophan that is in excess of current requirements will be fully metabolized in the liver. If there is ever a need to divert tryptophan metabolism from the liver to the periphery, especially to the immune system, then IDO would provide the necessary switch. We propose that strong IDO activation initiates a “kynurenine switch” that diverts a substantial proportion of downstream kynurenine pathway metabolism and NAD^+^ synthesis from the liver to the immune system (Figure 12). Quoting from our Quin-IR studies on tryptophan and kynurenine loading: “*During immune system activation, the metabolite in the kynurenine pathway that reaches the highest concentration appears to be kynurenine itself, and tissues such as lung are significant producers of kynurenine* (92). *If kynurenine is taken up poorly by hepatocytes and avidly by leukocytes, as our results suggest, then one function of the extensive activation of IDO in tissues such as lung may be to shift tryptophan utilization from the liver to the immune system”* (29). The strong induction of IDO in many tissues during an immune response has not been satisfactorily explained. We propose that a primary function is to shift NAD^+^ metabolism from centralized hepatic production to distributed immune system production. This local, on-demand substrate diversion for NAD^+^ production would clearly be more efficient than relying on hepatic supplies of nicotinamide. This view ties together many themes on tryptophan metabolism and has greater explanatory power than the tryptophan depletion hypothesis. We suggest that the tryptophan utilization model provides a unifying conceptual framework for understanding the multiple roles played by IDO, kynurenines, and Quin in regulating immune system responsiveness, tolerance, and metabolism in cells of the immune system.

Quin release and uptake for use in NAD^+^ synthesis is evolutionarily conserved in eukaryotes as it has been shown to occur in yeast. Quin is released by yeast cells and taken up by other yeast cells where it is converted to NAD^+^ (93). It is not known to what extent this occurs in mammals. In mammalian systems, it is more likely that, upon immune stimulation, various cell types release excess kynurenine, which is then taken up and converted to NAD^+^ by cells of the immune system. As such, IDO activation in cells such as lung cells does not “deplete tryptophan,” it initiates a biosynthetic pathway that generates excess systemic kynurenine, which can then be taken up and utilized preferentially by cells of the immune system.

In macrophages and other cells that utilize tryptophan or kynurenine to generate high internal concentrations of Quin, it is apparent that they slow or block further Quin utilization for at least two primary purposes, to stockpile an NAD^+^ precursor for later use, and to increase the concentration and release of upstream kynurenine metabolites to regulate other immune cell's responses. Interestingly, extracellular tryptophan can be exchanged with intracellular kynurenine across the plasma membrane in cells in culture via the large neutral amino acid transporter SLC7A5, also called LAT1 (94). SLC7A5 is also involved in kynurenine uptake and is expressed in T cells (95), B cells (96), and monocytes/macrophages (97), indicating that all of these cell types are able to take up extracellular kynurenine.

### Quin-IR in the Brain vs. Periphery

In humans, Quin is excreted in the urine and the level in urine increases with increasing oral doses of tryptophan (98). This suggests that when tryptophan availability is in excess of current needs, the kynurenine pathway provides an efficient means for metabolism and subsequent excretion via the kidneys (46). Quin levels rise in the bloodstream and in many tissues during infections and after stimulation with agents such as PWM (92), indicating that some cell types release Quin in response to pathogens or inflammatory signals. Both before and after PWM treatment, Quin levels are lowest in the brain and highest in the spleen (92), which is consistent with our current findings. Quin levels in normal mouse brain tissue are at detection limits by LC/MS-MS (99). In a comprehensive evaluation of the mechanisms controlling Quin levels in the brain, Morrison et al. showed that three primary factors maintained low brain concentrations. These included local synthesis, entry into the brain from the blood, and active Quin extrusion from brain by a probenecid-sensitive pump (100).

Quin is excluded from the brain to the extent possible, including during an immune response (28, 101). Quin levels in the brain are maintained below blood levels by barriers and transporters (100). During strong immune stimulation with LPS infusion into the brain, the extracellular Quin level, as measured by microdialysis, increased 66-fold (102). This was reportedly due to a combination of increased local synthesis and a decrease in the efflux/influx ratio. Normally, Quin synthesis in the brain is very low, but when super-physiological concentrations of 3-hydroxyanthranilate are provided, microglia produce significant quantities of Quin as shown by immunohistochemistry (31). During potent immune stimulation, Quin levels rise in many tissues, but brain levels remain low (92, 103). Further, in the current study, we induced a powerful and sustained immune response in the brain via LPS injection, and this only resulted in a minimal infiltration of Quin-positive cells (Figures 6–9). Peripheral PWM administration resulted in splenomegaly and a dramatic increase in Quin-IR in the spleen and liver (Figures 3, 4), but no immunoreactive cells were observed in brain parenchyma (Figure 2).

The low number of Quin-IR cells in the brain after direct LPS administration and subsequent local tissue necrosis is noteworthy. Lectin-stained macrophages and activated microglia outnumbered Quin-IR macrophages by many fold. Kynurenine metabolism in the brain is reported to be complete and robust under various conditions of immune stimulation, and in the case of LPS infusion into the brain, 98% of kynurenine and Quin in the brain was derived from local synthesis rather than from the blood supply (104). But as we have shown here, unlike peripheral tissues, Quin accumulation does not occur in the vast majority of cells even after direct LPS injection into the brain. In fact, the lack of Quin production within the area of damage may have contributed to the uncontrolled immune response and tissue necrosis noted by day 3 after LPS injection. If the kynurenine pathway response to immune stimulation in the brain is diminished, as our results suggest, then its immunosuppressive effects may be attenuated and less effective, resulting in more extensive tissue damage.

### Quin Accumulation in Cells of the Immune System

Because Quin is a neurotoxin, and has pro-apoptotic effects on some cell types, the accumulation of high intracellular levels in specific cells of the immune system is of significant interest. Numerous studies have shown that Quin accumulates in tissues and blood during infections or immune challenge, but very few methods allow researchers to look at Quin accumulation in various cell types throughout different tissues and organ systems. Quin immunohistochemistry provides a means to examine this question in detail. Immunohistochemistry is more useful at locating cells with high concentrations of the target molecule than it is at localizing trace amounts. Quin immunohistochemistry clearly shows that most cell types do not generate high intracellular levels. It is now clear that several immune system cell types are the most likely to accumulate substantial levels of cytosolic Quin including monocytes/macrophages, dendritic cells, microglia, Langerhans cells, and Kupffer cells, suggesting that phagocytes and antigen-presenting cells are major producers (26, 27, 31).

We have shown that Quin-IR is increased in a number of different conditions where the immune system is stimulated, in addition to those detailed here, including SIV in monkeys (105), human T-cell lymphotropic virus type 1 infection of human peripheral blood monocytes/macrophages (106), and in a murine AIDS model (107). Also, tryptophan and kynurenine loading in rats increased Quin-IR differentially in distinct cell types demonstrating selective uptake and utilization systems for these closely related metabolites (29). The plasma kynurenine to tryptophan ratio (Kyn/Trp) is often used as an indicator of IDO action and kynurenine pathway flux, despite its complications and limitations (65). Quin-IR provides a unique method for looking at kynurenine pathway throughput up to the point of Quin synthesis. Most of the active signaling metabolites are in the early segments of the kynurenine pathway initiated by IDO (see Figure 1). The metabolites in the remaining segments of the kynurenine pathway are destined for other metabolic fates, including NAD^+^ synthesis or oxidation for energy derivation.

**Figure 13:**
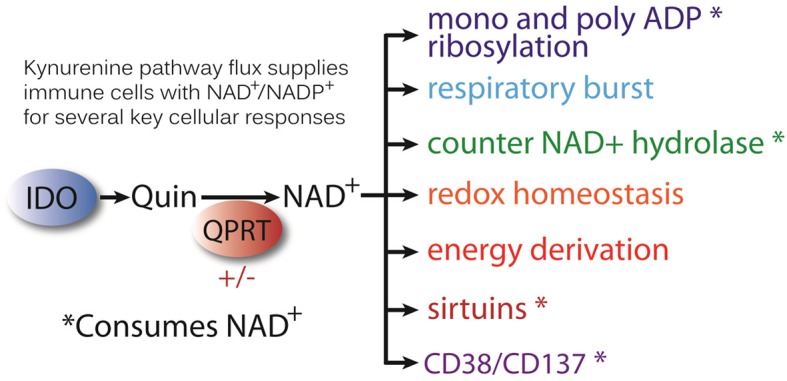
The kynurenine pathway generates immune modulatory agents, but it also supplies Quin to be directed to NAD^+^ synthesis in cells of the immune system. Regulation of the enzyme QPRT controls the flow of kynurenine pathway metabolism to NAD^+^. Increased NAD^+^ demand during inflammation and infection is driven by a number of cellular actions including energy derivation, the respiratory burst, and cellular redox homeostasis. In addition, there are a number of enzymatic reactions that use NAD^+^ as cosubstrate, and these reactions consume NAD^+^. These include the PARP reaction for DNA repair (poly-ADP ribosylation) and mono-ADP ribosylases, such as tankerases TNKS1 and 2. Increased NAD^+^ demand may also come from infections by bacteria that employ NAD^+^ hydrolases to deprive phagocytes of intracellular NAD^+^. NAD^+^ is also consumed by the NAD^+^-dependent deacetylases known as sirtuins, which regulate metabolism. Certain host enzymes/receptors also have NAD^+^ hydrolase activity including CD38 and CD157, which are ADP-ribosyl cyclases and hydrolases.

There could be many explanations for the observation that Quin is generated at high intracellular levels in certain immune cells. Foremost, halting the kynurenine pathway at QPRT and ACMSD in macrophages would result in stockpiling of Quin after strong IDO induction and allow for controlled release of upstream metabolites to modulate immune responses. It could also allow for sustained conversion to NAD^+^. Other potential reasons for Quin stockpiling include the fact that many bacteria secrete NAD^+^ glycohydrolases [e.g., tuberculosis necrotizing toxin or TNT, streptococcus pyogenes beta-NAD^+^(+) glycohydrolase, known as SPN, etc.] that deplete intracellular NAD^+^ levels in macrophages, leading to cell death (108–110). It is possible that some phagocytes accumulate Quin from tryptophan catabolism to replenish depleted NAD^+^ resulting from pathogens that secrete NAD^+^ hydrolase enzymes. In addition, endogenous NAD^+^ degrading enzymes such as CD38 (111) may also favor increased NAD^+^ synthesis from tryptophan. CD38 and CD157 are ectonucleotidase enzymes involved in immune tolerance that convert NAD^+^ into ADP-ribose and cyclic-ADP-ribose (112). Overall, the increased requirements for NAD^+^ synthesis during inflammation and infection include the PARP reaction, cell surface ADP ribosyltransferases, the respiratory burst, sirtuins, NAD^+^ hydrolase activity, ectonucleotidase activity, and maintaining redox homeostasis and energy derivation (Figure 13). All of these actions are enhanced during an immune response.

Most kynurenine pathway metabolites increase in blood plasma during infection or after administration of immune stimulants such as LPS, PWM, or INF-γ. Cells that have high levels of Quin are those that contain the enzymes of the kynurenine pathway up to QPRT and ACMSD (see Figure 1). It is likely that restriction at the level of QPRT is one reason why certain cells of the immune system show up strongly in Quin immunohistochemistry. QPRT activity is low in unstimulated monocytes, but is increased significantly after IFN-γ treatment (113). The Km of QPRT is 100 times greater than the normal levels of Quin in the liver, but the Vmax of the enzyme is low, meaning that during times of high tryptophan throughput, some degree of Quin accumulation would be expected (114). Therefore, when cells of the immune system are activated by cytokines such as INF-γ and IDO activity is greatly increased, the rapid tryptophan catabolism would be expected to cause some degree of Quin accumulation. High-dose administration of tryptophan in rats leads to more than a doubling of the Quin levels in the liver (114). We observed a modest increase in Quin-IR in the liver after high-dose tryptophan (29). The increase in Quin staining in liver after tryptophan or kynurenine administration indicates that Quin IHC can detect transiently increased levels in cells that express QPRT such as hepatocytes. Therefore, similar effects should be seen in leukocytes that further process Quin to NAD^+^, such as macrophages. Using Quin immunohistochemistry, several cell types showed mild to moderate levels of Quin staining, including B cells in splenic follicles after LPS injection into the hippocampus, and in gerbil hepatocytes after IP PWM injection.

### How Much NAD^+^ Is Synthesized From Quin Outside the Liver?

Adequate diets supply substantial amounts of NAD^+^ precursors including nicotinic acid and nicotinamide (collectively known as niacin). However, the tryptophan-to-NAD^+^ pathway operates independently of niacin supply (115). When tryptophan is required for NAD^+^ synthesis due to a poor diet or prolonged fasting, the liver activates TDO and tryptophan is catabolized through the kynurenine pathway to NAD^+^ (116). NAD^+^ is then broken down to nicotinamide and ADP ribose, and the nicotinamide is released to the circulation to be used by other cells throughout the body. However, less information is available on how much NAD^+^ is synthesized from Quin in cells of the immune system, especially during various immune responses and during the types of oxidative and metabolic stress that would be encountered during an injury or infection. Based on work with TDO-deficient mice and niacin-controlled diets, Terakata et al. proposed that the TDO-deficient mice could develop normally on a niacin-deficient diet because extra hepatic cells released kynurenine, which the liver took up and converted to Quin and then NAD^+^ (117). These findings highlight the fact that kynurenine synthesis via IDO and release from various cell types provides sufficient substrate for NAD^+^ synthesis to meet both developmental and nutritive needs. Kynurenine released to the bloodstream effectively bypasses the rate-limiting enzymes TDO and IDO in any cell type that is capable of kynurenine uptake (23, 29).

IDO, KMO and QPRT (see Figure 1) are all upregulated in human peripheral blood monocytes by IFN-γ (113), and this is consistent with the potential for extra hepatic NAD^+^ synthesis in macrophages. It was reported by Grant that INF-γ increases IDO and in turn increases NAD^+^ synthesis from tryptophan in RAW264.7 macrophages (13). In these studies, it was also shown that inhibition of the PARP reaction increased intracellular NAD^+^ levels by 35% over controls, whereas PARP inhibition along with INF-γ treatment resulted in an 84% increase in NAD^+^ content. Tryptophan was required for the effect and IDO inhibition reduced NAD^+^ levels. These findings indicate that the PARP reaction is a major utilizer of intracellular NAD^+^, and that IDO catabolism of tryptophan does replenish NAD^+^ supplies. NAD^+^ utilized for redox reactions is not consumed, but many enzyme systems such as the PARP reaction utilize NAD^+^ as cosubstrate and convert it to nicotinamide and ADP-ribose, thus depleting intracellular supplies (118). Recently, studies on NAD^+^ synthesis from tryptophan were repeated in primary human monocytes where it was found that induction of kynurenine metabolism by INF-γ was more robust in macrophages than in undifferentiated monocytes (14). INF-γ increased IDO activity as well as kynurenine and Quin levels in the culture media. Quin levels in the culture media after INF-γ treatment were an order of magnitude lower than the kynurenine levels, indicating that kynurenine is greatly favored over Quin for export from activated macrophages. Interestingly, the effect of INF-γ alone on human monocytes and macrophages was to cause a decrease in NAD^+^ levels because INF-γ also strongly activated PARP. However, a PARP inhibitor resulted in an increase in NAD^+^ levels in macrophages and INF-γ plus the PARP inhibitor further increased NAD^+^ levels (14).

In one of the first comprehensive studies of the contribution of the kynurenine pathway to *de novo* NAD^+^ synthesis, Minhas et al. investigated the importance of the IDO-mediated pathway in macrophages. They confirmed *de novo* NAD^+^ synthesis using mass labeled kynurenine, and showed that human monocyte-derived macrophages generated 40% of their NAD^+^ from kynurenine, rather than from the salvage pathway (15). Blockade of the salvage pathway resulted in over 90% of NAD^+^ synthesis coming from the kynurenine pathway. QPRT^−/−^ mice did not incorporate mass labeled tryptophan or kynurenine into NAD^+^. Loss of QPRT activity also affected mitochondrial oxidative phosphorylation in macrophages and reduced phagocytosis. A major finding by Minhas and coworkers was that LPS resulted in strong activation of the kynurenine pathway in macrophages, but that it also decreased QPRT expression, resulting in a bottleneck in NAD^+^ synthetic capacity. This maintained the macrophages in an inflammatory state with reduced phagocytosis. Overexpression of QPRT overcame the bottleneck and restored homeostasis. The authors suggested that QPRT represents a functional switch between pro-inflammatory and anti-inflammatory macrophage phenotypes.

Clearly, regulation of the expression and activity QPRT is the primary control point in the switch from Quin accumulation and release of kynurenines to NAD^+^ synthesis. As such, Quin immunohistochemistry provides a useful tool for determining which particular cell types are accumulating Quin during various immune challenges. It can be assumed that the Quin-IR cells have high IDO and relatively low QPRT activity. This may also explain the lack of Quin-IR in the vast majority of phagocytes in the brain after intracerebral LPS injection. During the transition from an active, inflammatory state to an anti-inflammatory, phagocytic state, macrophages upregulate QPRT activity and consume their intracellular Quin to restore their NAD^+^ supplies.

## Conclusions

Something interesting is going on with tryptophan during inflammatory and immune responses. It is now well-established that various kynurenine pathway metabolites are involved in regulating immune responses to various pathogens and pathologies, as well as acting to limit responses to self-antigens. The fact that the kynurenine pathway is evolutionarily conserved clearly indicates that it serves important biological functions. When this system works as intended, it confers survival advantages, but when it goes awry, there are substantial deleterious consequences. Maintaining this balance appears to involve a number of signaling systems, and the expression and regulation of various enzymes in the kynurenine pathway in different cell types. Further, diet may turn out to have an influence on the pro-survival benefits conferred by kynurenine pathway metabolism in the hepatic and immune systems, wherein excess protein in a calorie dense diet may exaggerate deleterious effects of strong kynurenine pathway activation and alter the course of inflammatory disorders or cancer. Humans require ~250 mg of tryptophan per day to maintain nitrogen balance, but modern diets rich in dairy products and meat contain 3–4 times as much or more (119). Interestingly, studies indicate that high protein diets have a negative impact on plasma NAD^+^ levels (120). It is possible that prolonged dietary tryptophan excess during a strong immune response may turn out to be an exacerbating factor in certain inflammatory conditions, shifting kynurenine pathway flux from a net positive to an overall negative effect. The effects of excessive kynurenine flux on cancer cell evasion may be another pathology associated with excess tryptophan in the diet (121).

Quin immunohistochemistry provides a unique window on kynurenine pathway throughput by highlighting cells that accumulate high intracellular levels of Quin. This intracellular stockpiling effect would not be apparent by most other analytical methods, but becomes obvious using Quin immunohistochemistry. Cells that are constitutively capable of synthesizing NAD^+^ from Quin such as hepatocytes do not demonstrate strong Quin stockpiling, but they do show a moderate, transient increase in Quin-IR after tryptophan loading or immune stimulation. In contrast, cells of the immune system including macrophages, dendritic cells, Langerhans cells, Kupffer cells, and others generate high, sustained levels of intracellular Quin in response to various immune stimulants. Because Quin is present at very high concentrations in the cytoplasm of these cells, they did not release all of their kynurenine metabolites, nor did they fully metabolize Quin to NAD^+^ or CO_2_. The most obvious conclusion is that intensely stained Quin-IR cells catabolized tryptophan through the pathway up to the point of QPRT and ACMSD. There is a bottleneck at these enzymatic steps, either via regulatory mechanisms that temporarily limit further metabolism of Quin, or enzyme saturation. Our initial impression on viewing this Quin stockpiling effect in 1994 was that it was associated with at least two functions: the production of immune signaling agents and increased NAD^+^ synthesis in the immune system (26). Further research will be required to fully answer the question of whether kynurenine pathway metabolism from kynurenine to NAD^+^ has a net positive or negative effect on outcomes in various pathological conditions. We expect that under many circumstances, the kynurenine-to-NAD^+^ pathway will turn out to be essential for host cell protection while also regulating the response to and resolution of various infectious or inflammatory conditions. We hypothesize that kynurenine metabolites will also turn out to facilitate or regulate the motility of certain immune cell types, including lamellipodia formation and movement through tissues, which could be important for connections between IDO, kynurenines, and cancer metastasis. Kynurenines may also be involved in regulating or facilitating phagocytosis. Overall, we predict that kynurenine pathway activation during immune responses to various pathogens and stimuli will turn out to be protective of cells in the immune system and facilitate pathogen clearance and resolution of the immune response. The balance between killing pathogens or cancer cells and sparing host cells is delicate, and the chances for dysregulation under physiological stress are significant. Most research is currently focused on the negative effects of activation of the kynurenine pathway via IDO, especially in the nervous system and in cancer. However, it is likely that research focused on the protective functions of this pathway, including the release of metabolites that modulate the interplay between immunogenic and tolerogenic responses, as well as the diversion of NAD^+^ metabolism from the liver to the immune cells and concomitant extra-hepatic production of NAD^+^, will provide further insights into the evolutionary conservation of this metabolic signaling pathway in the immune system.

## Data Availability Statement

The raw data supporting the conclusions of this article will be made available by the authors, without undue reservation, to any qualified researcher.

## Ethics Statement

All protocols were approved by the Uniformed Services University of the Health Sciences (USUHS) animal care and use committee.

## Author Contributions

JM, AN, and PA conceived the study and conducted the studies. JM, AN, PA, JI, NP, AB, and RV analyzed the data and wrote the paper.

### Conflict of Interest

The authors declare that the research was conducted in the absence of any commercial or financial relationships that could be construed as a potential conflict of interest.
