# Supplementary material for: Quinolinate as a Marker for Kynurenine Metabolite Formation and the Unresolved Question of NAD+ Synthesis During Inflammation and Infection
Source: Front Immunol. 2020 Feb 21;11:31. doi: 10.3389/fimmu.2020.00031 (PMC7047773; doi:10.3389/fimmu.2020.00031)
Supplement: Supplementary file 1 [file Data_Sheet_1.pdf]

Supplementary Material

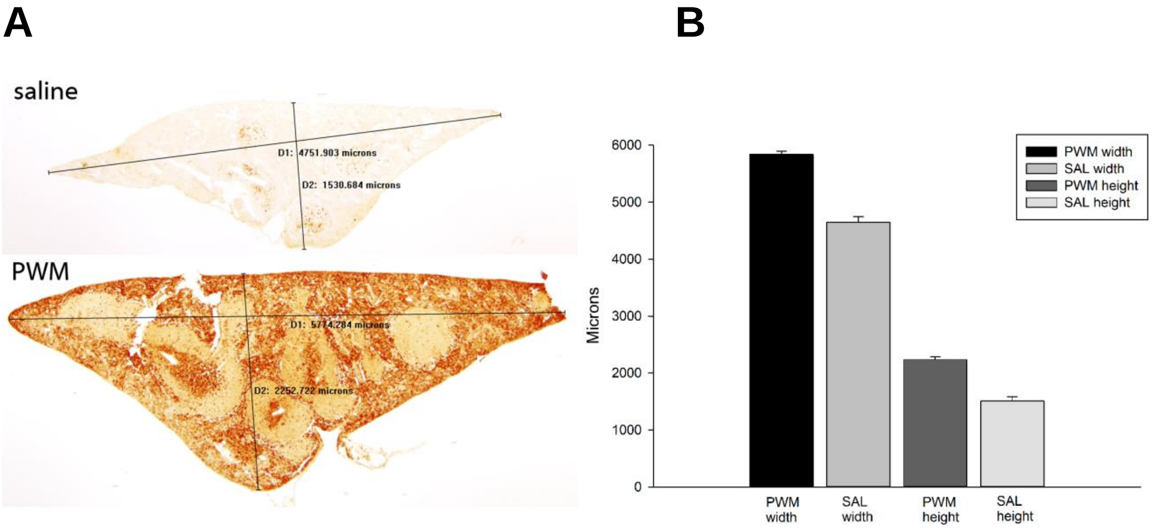

Supplementary Figure 1: PWM caused splenomegaly 24 hours after intraperitoneal administration as shown in these images stained for Quin (**A**). The splenomegaly was accompanied by a dramatic increase in Quin-IR in response to PWM. In cross sectional slices, the fulminant immune reaction resulted in a 25% increase in average section width and a 48% increase in average section height (**B:** average of 4 PWM and 5 saline spleen slices).

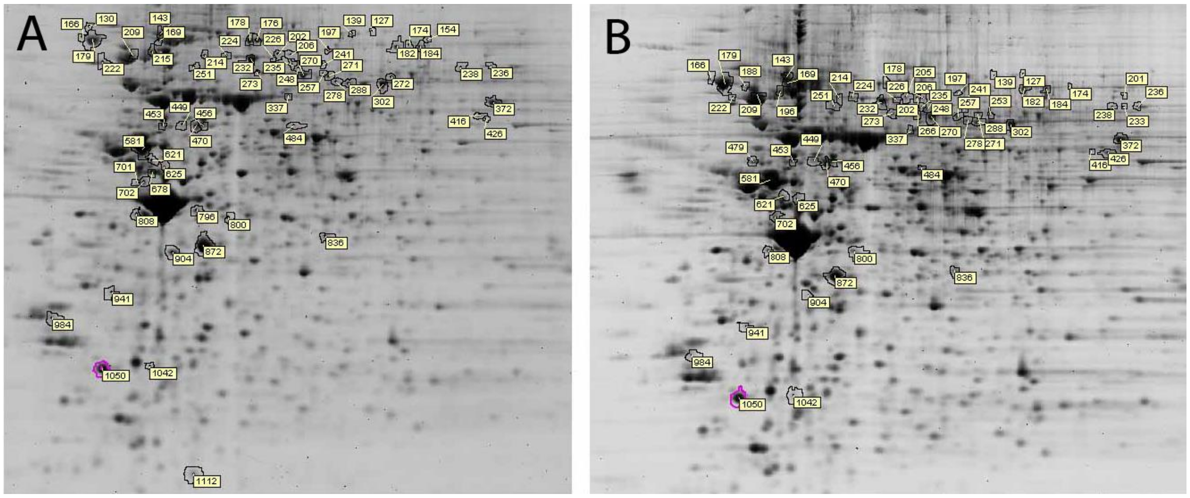

Supplementary Figure 2: 2D-DIGE gels with resolved spots numbered. Panel **A** shows the gel from control mice given saline, whereas panel **B** shows the gel from the mice administered 300 mg/kg kynurenine by IP injection 8 hours before sacrifice. N = 3 per group.
